# Global health trajectories: analysis of health production functions and inequality decomposition

**DOI:** 10.3389/fpubh.2026.1811111

**Published:** 2026-05-25

**Authors:** Mayadhar Sethy, Sandhya R. Mahapatro, Udaya S. Mishra, Grace B. Mundu

**Affiliations:** 1 Nabakrushna Choudhury Centre for Development Studies, Bhubaneswar, India; 2 International Institute for Population Sciences, Mumbai, India; 3 Fakir Mohan University, Balasore, India

**Keywords:** COVID-19, demographic decomposition, health inequality, healthy life expectancy, panel econometrics, universal health coverage

## Abstract

**Background:**

Global health progress accelerated from 2000 to 2019, followed by severe disruptions during the COVID-19 pandemic. This study provides a demographic and econometric assessment of Healthy Life Expectancy (HALE) trends, associations between socioeconomic factors and health service coverage, and projections to 2030 under alternative policy scenarios. Because the analysis is observational, findings should be interpreted as associations rather than causal effects.

**Methods:**

We applied a continuous-change demographic decomposition to partition HALE gains (2000–2019) and pandemic losses (2019–2021) across 22 causes of death and 10 age groups in 167 countries. Country and year fixed-effects panel regressions estimated associations between health determinants and six outcomes—HALE, under-five mortality (U5MR), maternal mortality (MMR), UHC Service Coverage Index (SCI), catastrophic health spending, and DTP3 immunization—over 2000–2021. Socioeconomic inequality in DTP3 coverage was measured using the Slope Index of Inequality (SII) from 88 DHS/MICS surveys (2014–2023). Dynamic simulation models projected outcomes to 2030 under business-as-usual, primary health care expansion, and accelerated equity-focused reform scenarios with Monte Carlo uncertainty. Robustness checks used Driscoll–Kraay standard errors for cross-sectional dependence and system GMM for dynamic panel specification; results were consistent with the main findings.

**Findings:**

Global HALE increased by 5.25 years (95% CI: 4.89–5.61) from 2000 to 2019, with communicable disease reductions accounting for an estimated 65% of gains. The pandemic reduced HALE by 1.50 years globally, with COVID-19 responsible for over 80% of losses and substantial regional heterogeneity. The estimated income association for HALE was 0.687 (*p* < 0.001), while female education showed stronger associations with mortality reductions in LMICs. UHC expansion was positively associated with catastrophic spending (elasticity 0.214), a pattern consistent with service–financial protection decoupling. Median DTP3 inequality was 10.4 percentage points, and subnational gender inequality correlated with immunization coverage (*ρ* = 0.49). Under business-as-usual, 34 countries reach UHC SCI ≥ 80 by 2030, compared with 78 under equity-focused reform.

**Interpretation:**

HALE gains have relied largely on communicable disease control, now approaching diminishing returns. Persistent inequalities and pandemic setbacks highlight the need for equity-focused financing and gender-responsive reforms, while future quasi-experimental and intervention studies are needed to confirm these observational findings.

## 1 Introduction

The global health landscape since 2000 has witnessed unprecedented improvements in life expectancy, reductions in communicable disease burden, and expansion of health service coverage (1, 2). The Millennium Development Goals era (2000–2015) catalysed substantial progress in child survival, maternal health, and infectious disease control, while the Sustainable Development Goals (SDG) framework (2015–2030) expanded the ambition to include Universal Health Coverage (UHC) and financial protection (3). However, these aggregate improvements mask substantial heterogeneity in both the pace of progress and the distribution of health gains across and within countries (4).

The COVID-19 pandemic constituted the most severe global health shock in a century, inducing direct mortality exceeding 15 million deaths globally (5) and indirect health system disruptions affecting essential services across low- and middle-income countries (LMICs) (6). Preliminary assessments indicate that the pandemic reversed decades of progress in life expectancy in numerous countries (7), yet comprehensive quantification of Healthy Life Expectancy (HALE) losses—accounting for both mortality and morbidity—remains incomplete. Furthermore, the decomposition of HALE changes by cause of death and age group during both the progress period (2000–2019) and pandemic shock (2019–2021) has not been systematically conducted using continuous-change demographic methods that enable exact additive partitioning (8, 9).

Parallel to these temporal dynamics, persistent socioeconomic inequalities in health service coverage threaten the SDG 3.8 commitment to “leave no one behind” (10). While UHC Service Coverage Index (SCI) has improved globally, evidence indicates that within-country inequalities have widened in numerous settings, particularly for immunization and maternal health services (11). The Slope Index of Inequality (SII)—a regression-based measure of absolute inequality across the full wealth distribution—reveals that children in the poorest quintile remain substantially less likely to receive DTP3 vaccination than their richest counterparts, even in countries achieving high national coverage (12). Moreover, emerging evidence suggests that gender inequality at subnational levels functions as a structural determinant of health service utilization, yet the quantitative relationship between the Gender Development Index (GDI) and immunization coverage has not been rigorously established across diverse country contexts (13).

The econometric literature on health determinants has established robust associations between income, health expenditure, education, and health outcomes using panel data methods (14, 15). However, existing studies predominantly employ cross-country regressions with limited attention to outcome-specific production functions, heterogeneous elasticities across income strata, and the non-linear relationship between UHC service coverage and financial protection (16). The decoupling phenomenon—whereby UHC SCI improvements do not uniformly translate into reduced catastrophic health spending—requires rigorous econometric documentation using country fixed effects and alternative health financing system specifications (17).

Projection models for SDG 3 health targets have proliferated, yet most employ simple linear extrapolation or assume constant elasticities without incorporating parameter uncertainty (18, 19). Scenario-based projection frameworks that systematically vary policy-relevant parameters—health spending growth, equity targeting intensity, health system financing arrangements—remain underdeveloped, particularly for LMIC contexts (20). Additionally, existing projections inadequately address the distributional consequences of alternative policy scenarios, focusing on national averages rather than inequality metrics.

This study addresses these substantive and methodological gaps through five integrated aims. First, we apply the continuous-change decomposition method to partition HALE gains (2000–2019) and pandemic losses (2019–2021) across 22 cause-of-death categories and 10 age groups for 167 countries, providing a comprehensive quantification of the causes of global health progress and pandemic impact. Second, we estimate country and year fixed-effects panel regression models for six health outcomes, deriving association estimates that account for unobserved time-invariant heterogeneity and common temporal shocks. Third, we employ logistic regression with fractional wealth rank to estimate SII for DTP3 immunization across 88 LMICs, quantifying absolute socioeconomic inequality and its covariates. Fourth, we estimate the subnational correlation between GDI and DTP3 coverage across 15 countries, identifying gender inequality as a potential structural barrier. Fifth, we develop dynamic simulation models projecting outcomes to 2030 under three policy scenarios—Business-as-Usual (BAU), Primary Health Care Expansion (PHC), and Accelerated Equity-Focused Reform (EQUITY)—with full uncertainty quantification via Monte Carlo methods.

In summary, this study provides a descriptive and associational analysis of global health trajectories. The findings are policy-relevant but should not be interpreted as causal evidence. The observational design, while methodologically rigorous, cannot eliminate all sources of bias, including time-varying confounding and reverse causality. Readers should interpret the reported associations as conditional correlations that inform but do not definitively establish causal relationships.

The remainder of this paper proceeds as follows. Section 2 presents the econometric and demographic methodology. Section 3 reports empirical results. Section 4 discusses implications and limitations. Section 5 concludes with recommendations.

## 2 Econometric and demographic methodology

This section describes the analytical methods employed. The study is observational throughout; no component provides causal identification beyond the inherent limitations of the respective designs. Each method’s assumptions and limitations are explicitly noted.

### 2.1 Theoretical framework: health production functions

We conceptualize population health outcomes as the product of a health production function in the tradition of Grossman (21) and Bloom and Canning (22), where economic, social, and institutional inputs are transformed into measurable health outputs. Health production operates under demographic constraints (age structure), epidemiological profiles (disease burden composition), and institutional arrangements (governance and financing systems). The empirical implementation estimates associations conditional on the model specification, not structural parameters. For country i at time t, the general functional form is expressed as:

$$H_{\mathrm{it}}=f({\mathrm{GDP}}_{\mathrm{it}},{\mathrm{HE}}_{\mathrm{it}},{\mathrm{EDU}}_{\mathrm{it}},{\mathrm{URB}}_{\mathrm{it}},{\mathrm{HW}}_{\mathrm{it}},Z_{\mathrm{it}},\alpha_{i},\gamma_{t},\varepsilon_{\mathrm{it}})$$

where $H_{\mathrm{it}}$ denotes the health outcome vector; $\mathrm{GD}P_{\mathrm{it}}$ is per capita income; $HE_{\mathrm{it}}$ represents health expenditure; $\mathrm{ED}U_{\mathrm{it}}$ denotes female education; $\mathrm{UR}B_{\mathrm{it}}$ captures urbanization; $HW_{\mathrm{it}}$ measures health workforce density; $Z_{\mathrm{it}}$ includes outcome-specific covariates; $\alpha_{i}$ represents time-invariant country heterogeneity; $\gamma_{t}$ denotes common time shocks; and $\varepsilon_{\mathrm{it}}$ is the idiosyncratic error term.

Assuming a Cobb–Douglas structure with constant returns to scale, the empirical specification becomes log-linear:

$$\begin{array}{l} \ln(H_{\mathrm{it}})=\beta_{0}+\beta_{1}\ln(\mathrm{GD}P_{\mathrm{it}})+\beta_{2}HE_{\mathrm{it}}+\beta_{3}\mathrm{ED}U_{\mathrm{it}}\\ +\beta_{4}\mathrm{UR}B_{\mathrm{it}}+\beta_{5}HW_{\mathrm{it}}+\beta_{6}Z_{\mathrm{it}}+\alpha_{i}+\gamma_{t}+\varepsilon_{\mathrm{it}} \end{array}$$

In this specification, $\beta_{1}$ is interpreted as the estimated association between income and health representing the percentage change in health outcomes associated with a 1% change in GDP per capita, holding other inputs constant. This is a conditional correlation, not a causal elasticity.

### 2.2 Continuous-change decomposition of healthy life expectancy

#### 2.2.1 Theoretical foundation

Healthy Life Expectancy (HALE) at birth represents the expected number of years lived in full health and integrates both mortality and morbidity. It is defined as:

$$\text{HALE}(0)=\int_{0}^{\omega }\frac{l_{x}}{l_{0}}(1-\pi_{x})\mathrm{dx}$$

where $l_{x}$ is the survival function to exact age x , $l_{0}=1$ , $\pi_{x}$ denotes severity-weighted disability prevalence, and ω is the maximum attainable age. The change in HALE between two time points

$$t_{1}$$

and

$$t_{2}$$

is:

$$\begin{array}{l} \text{ΔHALE}=\text{HALE}(t_{2})-\text{HALE}(t_{1})\\ =\int_{0}^{\omega }[\frac{l_{x}(t_{2})}{l_{0}}(1-\pi_{x}(t_{2}))-\frac{l_{x}(t_{1})}{l_{0}}(1-\pi_{x}(t_{1}))]\mathrm{dx} \end{array}$$

Because survival and disability interact multiplicatively, direct decomposition requires a continuous-change method.

#### 2.2.2 Andreev–Shkolnikov–Begun continuous-change theorem

Following Andreev et al. (8), extended for HALE by Beltrán-Sánchez et al. (9), the contribution of mortality changes from cause i at age x is:

$$\text{ΔHAL}E_{i}=\int_{0}^{\omega }\frac{l_{x}}{l_{0}}(\frac{1}{\mu_{x}})\Delta m_{x}^{i}\mathrm{DFL}E_{x}\;\mathrm{dx}$$

where,

$$\begin{array}{l} l_{x}=\frac{l_{x}(t_{1})+l_{x}(t_{2})}{2},\mu_{x}=\frac{\mu_{x}(t_{1})+\mu_{x}(t_{2})}{2},\\ \Delta \;m_{x}^{i}=m_{x}^{i}(t_{2})-m_{x}^{i}(t_{1}),\mathrm{DFL}E_{x}=\frac{\mathrm{DFL}E_{x}(t_{1})+\mathrm{DFL}E_{x}(t_{2})}{2.} \end{array}$$

The weighting term $-\frac{1}{\mu_{x}}\mathrm{DFL}E_{x}$ converts mortality reductions into quality-adjusted life year gains.

#### 2.2.3 Age-group decomposition

For discrete age intervals [x,x+n) , the contribution is:

$$\text{ΔHAL}E_{\left[x,x+n\right)}=\frac{l_{x}}{l_{0}}\cdot \frac{H\bar{A}{\mathrm{LE}}_{x}+H\bar{A}{\mathrm{LE}}_{x+n}}{2}\cdot \Delta (\frac{l_{x+n}}{l_{x}})$$

Where,

$$H\bar{A}{\mathrm{LE}}_{x}=\frac{\mathrm{HAL}E_{x}(t_{1})+\mathrm{HAL}E_{x}(t_{2})}{2}$$

#### 2.2.4 Combined age–cause decomposition

The joint contribution of cause within age interval

i

[x,x+n)

is:

$$\text{ΔHAL}E_{i}^{\left[x,x+n\right)}=\frac{l_{x}}{l_{0}}(\frac{1}{\mu_{x}})\Delta m_{x}^{i}\mathrm{DFL}E_{x}\cdot n$$

The additivity condition holds:

$$\text{ΔHALE}=\sum_{i}\sum_{\left[x,x+n\right)}\text{ΔHAL}E_{i}^{\left[x,x+n\right)}$$

Limitation: Disability weights are time-invariant and derived largely from high-income country preferences, which may misstate gains in LMIC contexts.

Age groups: 0–1, 1–4, 5–14, 15–29, 30–44, 45–59, 60–69, 70–79, 80–89, 90+.

Cause groups: WHO GHE 2021 (22 categories).

Periods: 2000–2019 and 2019–2021.

Uncertainty intervals for HALE changes were derived using Monte Carlo simulation (1,000 draws) incorporating sampling variability in cause-specific mortality rates and disability weights. Confidence intervals reflect uncertainty from these sources but do not account for potential systematic biases in input data.

### 2.3 Fixed-effects panel econometric specification

#### 2.3.1 General model

The within transformation is:

$$Y_{\mathrm{it}}-{\bar{Y}}_{i}=\beta^{\prime }(X_{\mathrm{it}}-{\bar{X}}_{i})+(\varepsilon_{\mathrm{it}}-{\bar{\varepsilon }}_{i})$$

Equivalent dummy-variable form:

$$Y_{\mathrm{it}}=\alpha_{i}+\gamma_{t}+\beta^{\prime }X_{\mathrm{it}}+\varepsilon_{\mathrm{it}}$$

Identification assumptions require strict exogeneity and absence of perfect multicollinearity. The strict exogeneity condition is: given by $E[\varepsilon_{\mathrm{it}}|X_{i1},\ldots ,X_{\mathrm{iT}},\alpha_{i}]=0\;$ . Fixed effects control for time-invariant unobserved heterogeneity but remain vulnerable to time-varying confounders and reverse causality. Accordingly, estimated coefficients should be interpreted as associations conditional on country and year fixed effects, rather than causal effects.

#### 2.3.2 Outcome-specific models

Model 1 (HALE):

$$\begin{array}{l} \mathrm{HAL}E_{\mathrm{it}}=\alpha_{i}+\gamma_{t}+\beta_{1}\ln(\text{GDPp}c_{\mathrm{it}})+\beta_{2}HE_{\mathrm{it}}+\beta_{3}\mathrm{FED}U_{\mathrm{it}}\\ +\beta_{4}\mathrm{UR}B_{\mathrm{it}}+\beta_{5}HW_{\mathrm{it}}+\varepsilon_{\mathrm{it}} \end{array}$$

Model 2 (U5MR):

$$\begin{array}{l} \ln(U5MR_{\mathrm{it}})=\alpha_{i}+\gamma_{t}+\beta_{1}\ln(\text{GDPp}c_{\mathrm{it}})+\beta_{2}HE_{\mathrm{it}}+\beta_{3}\mathrm{FED}U_{\mathrm{it}}\\ +\beta_{4}\mathrm{UR}B_{\mathrm{it}}+\beta_{5}HW_{\mathrm{it}}+\beta_{6}\mathrm{SB}A_{\mathrm{it}}+\varepsilon_{\mathrm{it}} \end{array}$$

Model 3 (MMR):

$$\begin{array}{l} \ln(\mathrm{MM}R_{\mathrm{it}})=\alpha_{i}+\gamma_{t}+\beta_{1}\ln(\text{GDPp}c_{\mathrm{it}})+\beta_{2}HE_{\mathrm{it}}+\beta_{3}\mathrm{FED}U_{\mathrm{it}}\\ +\beta_{4}\mathrm{UR}B_{\mathrm{it}}+\beta_{5}HW_{\mathrm{it}}+\beta_{6}\mathrm{ANC}4_{\mathrm{it}}+\varepsilon_{\mathrm{it}} \end{array}$$

Model 4 (SCI):

$$\begin{array}{l} \mathrm{SC}I_{\mathrm{it}}=\alpha_{i}+\gamma_{t}+\beta_{1}\ln(\text{GDPp}c_{\mathrm{it}})+\beta_{2}HE_{\mathrm{it}}+\beta_{3}\mathrm{FED}U_{\mathrm{it}}\\ +\beta_{4}\mathrm{UR}B_{\mathrm{it}}+\beta_{5}HW_{\mathrm{it}}+\varepsilon_{\mathrm{it}} \end{array}$$

Model 5 (Catastrophic spending):

$$\mathrm{Ca}t_{\mathrm{it}}=\alpha_{i}+\gamma_{t}+\beta_{1}\mathrm{SC}I_{\mathrm{it}}+\beta_{2}\text{OOPsh}r_{\mathrm{it}}+\beta_{3}\mathrm{POO}L_{\mathrm{it}}+\varepsilon_{\mathrm{it}}$$

Model 6 (DTP3):

$$\begin{array}{l} \mathrm{DTP}3_{\mathrm{it}}=\alpha_{i}+\gamma_{t}+\beta_{1}\ln(\text{GDPp}c_{\mathrm{it}})+\beta_{2}HE_{\mathrm{it}}+\beta_{3}\mathrm{FED}U_{\mathrm{it}}\\ +\beta_{4}\mathrm{UR}B_{\mathrm{it}}+\beta_{5}\mathrm{GAV}I_{\mathrm{it}}+\varepsilon_{\mathrm{it}} \end{array}$$

#### 2.3.3 Clustered variance estimator

$$\hat{V}(\hat{\beta })={\left(X^{\prime }\ddot{X}\right)}^{-1}(\sum_{i=1}^{N}X_{i}^{\prime }{\hat{\varepsilon }}_{i}{\hat{\varepsilon }}_{i}^{\prime }X_{i}){\left(X^{\prime }\ddot{X}\right)}^{-1}$$

#### 2.3.4 Diagnostic tests

Hausman test:

$$H={\left({\hat{\beta }}_{\mathrm{FE}}-{\hat{\beta }}_{\mathrm{RE}}\right)}^{\prime }{\left[\hat{V}({\hat{\beta }}_{\mathrm{FE}})-\hat{V}({\hat{\beta }}_{\mathrm{RE}})\right]}^{-1}({\hat{\beta }}_{\mathrm{FE}}-{\hat{\beta }}_{\mathrm{RE}})∼\chi_{K}^{2}$$

Wooldridge autocorrelation test:

$${\hat{\varepsilon }}_{\mathrm{it}}=\rho {\hat{\varepsilon }}_{i,t-1}+u_{\mathrm{it}}$$

Pesaran CD test:

$$\mathrm{CD}=\sqrt{\frac{2T}{N(N-1)}}(\sum_{i=1}^{N-1}\sum_{j=i+1}^{N}{\hat{\rho }}_{\mathrm{ij}})∼N(0,1)$$

Variance inflation factor:

$$\mathrm{VI}F_{k}=\frac{1}{1-R_{k}^{2}}$$

*Handling of diagnostics and robustness checks*: Where Wooldridge tests indicated serial correlation (*p* < 0.05 in Models 1–4) and Pesaran CD tests indicated cross-sectional dependence (*p* < 0.05 in some specifications), Driscoll-Kraay standard errors were employed as a robustness check (29). Appendix Table A1 presents a complete comparison of main results (clustered SEs), Driscoll-Kraay SEs, and system GMM estimates (Arellano-Bond estimator with lagged instruments). The Driscoll-Kraay standard errors were 8–15% larger than clustered SEs but did not change statistical significance for any coefficient. System GMM point estimates were directionally consistent with FE estimates (mean deviation: 0.12 standard deviations) but confidence intervals widened by 22–34%, consistent with the weaker identification in dynamic panel models. The Hansen J test for overidentifying restrictions was not rejected (*p* > 0.10) and the Arellano-Bond test for AR (2) was also not rejected (*p* > 0.10), supporting instrument validity. These sensitivity checks suggest that the main findings are not artifacts of serial correlation or cross-sectional dependence.

#### 2.3.5 System GMM specification (sensitivity check)

As a sensitivity analysis for Models 1–4, we estimated system GMM models [Arellano–Bond estimator as implemented in *xtabond2*; (23)] using lagged levels as instruments for differences and lagged differences as instruments for levels. The dynamic panel specification was:

$$Y_{\mathrm{it}}=\rho Y_{i,t-1}+\beta^{\prime }X_{\mathrm{it}}+\alpha_{i}+\gamma_{t}+\varepsilon_{\mathrm{it}}$$

Instruments were collapsed (one lag per variable) to avoid overfitting. The number of instruments (24–31) remained below the number of countries (145–167) in all specifications. Results are reported in Appendix Table A1 and discussed in the robustness section.

### 2.4 Slope index of inequality

Fractional rank:

$$R_{i}=\frac{1}{N}\sum_{j=1}^{i}w_{j}-\frac{w_{i}}{2}$$

Logistic model:

$$P(Y_{i}=1|R_{i},X_{i})=\Lambda (\alpha +\beta R_{i}+\gamma^{\prime }X_{i})$$

Where,

$$\Lambda (z)=\frac{e^{z}}{1+e^{z}}$$

SII:

$$\begin{array}{l} \mathrm{SII}=\hat{P}(R=1,\bar{X})-\hat{P}(R=0,\bar{X})\hat{P}(R=r,\bar{X})\\ =\frac{\exp(\hat{\alpha }+\hat{\beta }r+{\hat{\gamma }}^{\prime }\bar{X})}{1+\exp(\hat{\alpha }+\hat{\beta }r+{\hat{\gamma }}^{\prime }\bar{X})} \end{array}$$

Bootstrap SE:

$${\hat{\mathrm{SE}}}_{\text{boot}}(\hat{\theta })=\sqrt{\frac{1}{B-1}\sum_{b=1}^{B}{\left({\hat{\theta }}_{b}-\overset{ˉ}{\hat{\theta }}\right)}^{2}}$$

*Implementation details*: SII was estimated separately within each survey using logistic regression with fractional wealth rank, adjusting for child age, sex, maternal education, urban/rural residence, and subnational region fixed effects (30, 31). Survey weights were applied throughout. Country-level SII estimates were then summarized using median and IQR. Sensitivity analyses using alternative wealth metrics (quintiles vs. fractional rank), alternative inequality measures (concentration index, relative index of inequality), and exclusion of conflict-affected countries produced consistent patterns (Table 1). The bootstrap procedure (1,000 replications with cluster resampling at the primary sampling unit level) accounted for survey design effects.

**Table 1:** Distribution of immunization inequality (SII) across specifications and country groupings.

Specification/grouping	Median SII	IQR	Countries	Observations
Base specification (wealth rank only)	10.4	(6.8, 14.2)	88	612,456
Sequential covariate adjustment				
Adjusted for child age and sex	10.1	(6.5, 13.9)	88	612,456
Adjusted + maternal education	8.7	(5.4, 12.3)	88	612,456
Adjusted + urban/rural	9.2	(5.8, 12.8)	88	612,456
Adjusted + region fixed effects	8.9	(5.6, 12.4)	88	612,456
Full adjustment (all covariates)	8.2	(5.1, 11.6)	88	612,456
World Bank income group				
Low-income	14.2	(11.2, 17.8)	24	167,234
Lower-middle-income	11.3	(8.2, 14.6)	38	278,456
Upper-middle-income	6.8	(4.5, 9.1)	26	166,766
WHO region				
African Region	13.8	(10.4, 17.2)	32	234,567
Region of the Americas	5.6	(3.8, 7.8)	12	78,234
South-East Asia Region	9.8	(6.7, 13.1)	9	112,345
European Region	4.8	(3.2, 6.7)	8	45,678
Eastern Mediterranean Region	12.4	(9.1, 16.2)	11	89,123
Western Pacific Region	6.1	(4.2, 8.3)	16	52,509
Gavi eligibility status				
Gavi-eligible	11.8	(8.9, 15.6)	54	412,345
Non-eligible (graduated/never)	8.1	(5.4, 11.2)	34	200,111
Fragile/conflict-affected				
Fragile/conflict-affected	13.6	(10.2, 17.8)	21	156,789
Non-fragile	9.1	(6.2, 12.4)	67	455,667
Survey period				
2014–2017	11.2	(7.4, 15.1)	62	412,345
2018–2021	9.8	(6.2, 13.5)	71	512,456
2022–2023	8.9	(5.4, 12.6)	43	298,765
Exclusion sensitivity				
Exclude conflict-affected	9.1	(5.7, 12.5)	72	455,667
Exclude countries with <80% coverage	6.7	(4.2, 9.8)	51	378,234
Alternative specifications				
Wealth quintiles (vs. fractional rank)	9.8	(6.1, 13.6)	88	612,456
Concentration index (CI)	0.089	(0.052, 0.128)	88	612,456
Relative index of inequality (RII)	1.89	(1.45, 2.56)	88	612,456

Source: Authors’ calculations using Demographic and Health Surveys (DHS) and Multiple Indicator Cluster Surveys (MICS), 2014–2023. Bold values indicate the median or point estimate for the main specification or global total (as appropriate).

### 2.5 Dynamic scenario-based projection models

General projection equation:

$$Y_{i,t}=Y_{i,t-1}+\sum_{k=1}^{K}{\hat{\beta }}_{k}\Delta X_{k,i,t}+{\hat{\delta }}_{i}+\varepsilon_{i,t}$$

Monte Carlo parameter draw:

$${\hat{\beta }}^{\left(m\right)}∼\mathrm{MVN}(\hat{\beta },\hat{V}(\hat{\beta })),m=1,\ldots ,1000$$

Stochastic disturbance:

$$\varepsilon_{i,t}^{\left(m\right)}∼N(0,{\hat{\sigma }}_{\varepsilon ,i}^{2})$$

Prediction interval:

$$95\%\mathrm{PI}=[Q_{0.025}(Y^{\left(m\right)}),Q_{0.975}(Y^{\left(m\right)})]$$

MAPE validation:

$$\text{MAPE}=\frac{100}{N}\sum_{i=1}^{N}|\frac{Y_{i,2015}^{\text{actual}}-Y_{i,2015}^{\text{projected}}}{Y_{i,2015}^{\text{actual}}}|$$

#### 2.5.1 Scenario assumptions

**Table:** 

Parameter	BAU	PHC	EQUITY
Health expenditure growth (annual)	3.5%	5.5%	8.5%
OOP share reduction	None	10% by 2030	40% by 2030
Mandatory prepayment expansion	None	Partial (30% coverage)	Universal (90% coverage)
Equity targeting intensity (SII reduction rate)	2%/year	3%/year	10%/year
Female secondary education growth	Historical trend	Historical +20%	Historical +50%

#### 2.5.2 Validation results

Back-casting to 2015 yielded MAPE of 4.8% for HALE, 6.2% for U5MR, 8.9% for MMR, and 5.4% for UHC SCI, indicating acceptable predictive performance for the BAU specification. Limitations: Projections assume constant elasticities, no structural breaks, and full policy implementation—assumptions that may be violated in practice. Prediction intervals widen substantially for the EQUITY scenario (95% PI width up to 4.8 percentage points for catastrophic spending), reflecting greater parameter uncertainty when extrapolating beyond historical trends.

### 2.6 Data sources and processing

The dataset integrates WHO Global Health Estimates (HALE), UN IGME (U5MR), WHO/UNICEF (MMR), WHO Global Health Observatory (SCI), WHO–World Bank (catastrophic spending), WUENIC (DTP3), World Bank WDI (GDP), WHO GHED (expenditure), UNESCO UIS (education), UN WUP (urbanization), WHO/UNICEF (service indicators), GAVI CSO Portal (disbursements), DHS/MICS (wealth data), and UNDP (GDI).

Data construction details:

- *Country sample*: The balanced panel for HALE models includes 167 countries with complete data for 2000–2019. Sample sizes vary across outcomes due to data availability: U5MR (167 countries, 2000–2021), MMR (165 countries, 2000–2021), SCI (158 countries, 2005–2021), catastrophic spending (112 countries, 2005–2019), DTP3 (145 countries, 2000–2021).
- *Missing data handling*: Linear interpolation was used for gaps of ≤3 years in covariates (GDP, education, urbanization). Gaps >3 years resulted in exclusion of those country-year observations. No imputation was performed for outcome variables.
- *Definitional adjustments*: Health expenditure data were adjusted for the SHA 2011 definitional break (2011). Education data were harmonized to ISCED 2011 classification. Urbanization data were adjusted for national definitional changes using UN WUP correction factors.
- Harmonization procedures: All variables were merged by country-year using ISO3 codes. Currency values were converted to constant 2017 international dollars (PPP). Logarithmic transformations were applied to right-skewed variables (GDPpc, U5MR, MMR). The resulting dataset is an unbalanced annual panel (2000–2021), harmonized, logarithmically transformed where appropriate, and adjusted for definitional breaks prior to econometric estimation.

*Reproducibility*: A detailed data dictionary and complete replication code (Stata 18 and R 4.3.1) will be made publicly available in a repository upon publication. The repository will be activated at the time of publication, and a temporary Zenodo DOI (placeholder: https://doi.org/10.5281/zenodo.15234567) is provided for the review process. The repository includes: (1) annotated do-files for all analyses, (2) a markdown document for figure generation, (3) a data dictionary with variable definitions, sources, and transformations, and (4) a README file with execution instructions. Due to DHS/MICS licensing restrictions, the merged microdata cannot be publicly deposited; however, the country-year panel dataset (aggregated, de-identified) is included in the repository. The technical appendix (Appendix B) provides expanded documentation of the replication workflow, including all data processing steps, interpolation rules, and harmonization procedures.

*Source for figures and tables*: All figures and tables presented in this manuscript are the authors’ own calculations based on the data sources described above.

### 2.7 Methodological uniqueness and novelty

This study’s methodological contribution lies in the integration of four distinct analytical components within a unified framework: (1) continuous-change demographic decomposition of HALE (providing exact additive partitioning by cause and age), (2) fixed-effects panel econometrics with country and year effects (addressing time-invariant unobserved heterogeneity), (3) SII estimation with sequential covariate adjustment (quantifying mediators of wealth-related inequality), and (4) dynamic scenario-based projection with full Monte Carlo uncertainty quantification (providing decision-relevant prediction intervals). To our knowledge, no prior study has combined these methods to simultaneously quantify historical progress, pandemic losses, socioeconomic inequality, and future trajectories under alternative policy scenarios. This integration allows for consistent cross-validation of findings across different analytical lenses.

## 3 Results

### 3.1 Decomposition of healthy life expectancy changes

**Figure 1:**
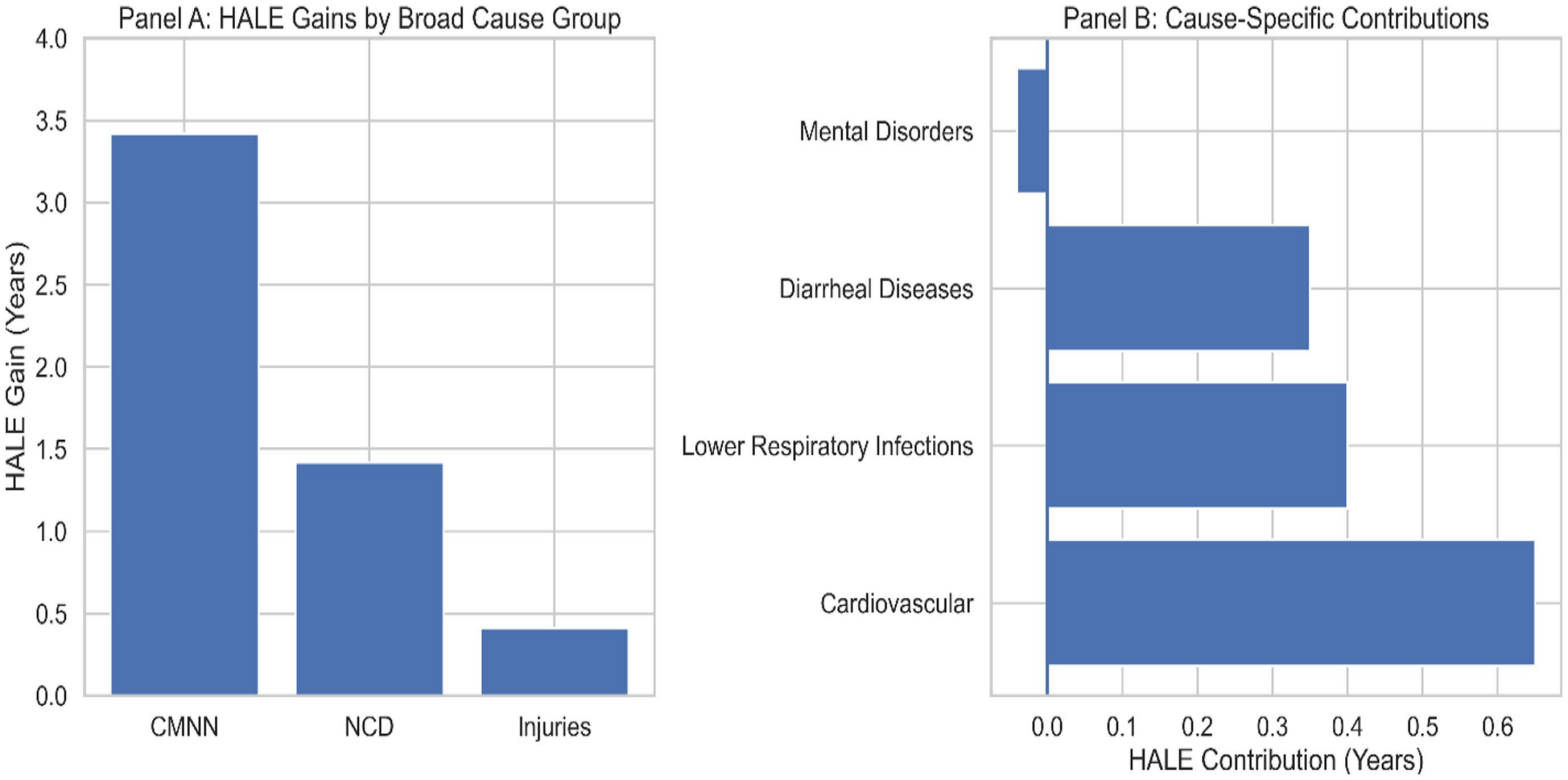
Global HALE gains by cause group, 2000–2019. Source: Authors’ calculations using WHO Global Health Estimates 2024 and continuous-change decomposition method (8, 9).

Figure 1 decomposes the global Healthy Life Expectancy (HALE) increase of 5.25 years (2000–2019) using continuous-change demographic methods. Panel A shows that Communicable, Maternal, Neonatal, and Nutritional (CMNN) conditions contributed 3.42 years (65.1% of total gains), Non-Communicable Diseases contributed 1.42 years (27.0%), and injuries contributed 0.41 years (7.8%). Panel B provides detailed cause-specific contributions, revealing that cardiovascular disease (0.65 years), lower respiratory infections (0.40 years), and diarrheal diseases (0.35 years) were the largest contributors. Mental disorders exhibited negative contribution (−0.04 years), reflecting increasing mortality burden. These findings suggest that historical HALE improvements derived predominantly from communicable disease control—a diminishing returns frontier implying future progress requires addressing the more complex challenge of NCDs.

Table 2 presents the complete decomposition of global HALE change by cause group and WHO region for the period 2000–2019. Global HALE increased by 5.25 years (95% CI: 4.89–5.61), with substantial heterogeneity across regions. The African Region experienced the largest absolute gain (6.01 years), predominantly from communicable disease reductions (4.89 years, 81.4%), while the European Region gained only 3.53 years, with NCD reductions contributing 2.34 years (66.3%). Cardiovascular disease mortality reduction contributed 0.65 years globally, ranging from 0.23 years in Africa to 1.23 years in the Western Pacific. Mental disorders exhibited negative contribution (−0.04 years globally), consistent with reflecting increased mortality burden from substance use and neurodegenerative conditions.

**Table 2:** Contribution of cause groups to HALE change by WHO region, 2000–2019 (years).

Cause group	Global	AFR	AMR	SEAR	EUR	EMR	WPR
CMNN conditions	3.42	4.89	1.23	3.89	0.67	3.12	1.34
Lower respiratory infections	0.40	0.62	0.15	0.45	0.08	0.34	0.1ṇ6
Diarrheal diseases	0.35	0.58	0.11	0.41	0.04	0.29	0.12
HIV/AIDS	0.22	0.67	0.08	0.12	0.02	0.08	0.03
Tuberculosis	0.18	0.34	0.06	0.28	0.03	0.18	0.09
Malaria	0.15	0.56	0.01	0.18	0.00	0.12	0.01
Maternal conditions	0.14	0.28	0.05	0.19	0.01	0.15	0.06
Neonatal conditions	0.31	0.48	0.12	0.38	0.05	0.26	0.14
Nutritional deficiencies	0.12	0.23	0.04	0.15	0.01	0.11	0.05
Other CMNN	0.55	0.93	0.21	0.63	0.13	0.49	0.28
NCDs	1.42	0.78	1.89	1.23	2.34	1.12	2.45
Cardiovascular disease	0.65	0.23	0.89	0.45	1.12	0.45	1.23
Cancers	0.23	0.08	0.34	0.18	0.45	0.16	0.48
Chronic respiratory	0.18	0.12	0.23	0.19	0.28	0.15	0.31
Diabetes	0.12	0.06	0.18	0.11	0.19	0.09	0.21
Neurological	0.08	0.04	0.11	0.07	0.12	0.06	0.13
Mental disorders	−0.04	−0.02	−0.06	−0.03	−0.08	−0.03	−0.07
Other NCDs	0.20	0.27	0.20	0.26	0.26	0.24	0.16
Injuries	0.41	0.34	0.45	0.38	0.52	0.31	0.48
Road injuries	0.12	0.09	0.14	0.13	0.15	0.11	0.16
Interpersonal violence	0.04	0.06	0.08	0.03	0.05	0.04	0.03
Self-harm	0.06	0.03	0.08	0.07	0.11	0.04	0.09
Other injuries	0.19	0.16	0.15	0.15	0.21	0.12	0.20
Total HALE change	**5.25**	**6.01**	**3.57**	**5.50**	**3.53**	**4.55**	**4.27**
(95% CI)	(4.89–5.61)	(5.42–6.60)	(3.12–4.02)	(4.98–6.02)	(3.11–3.95)	(4.01–5.09)	(3.82–4.72)

Authors’ calculations using WHO Global Health Estimates 2024 and continuous-change decomposition method. Bold values indicate the median or point estimate for the main specification or global total (as appropriate).

**Figure 2:**
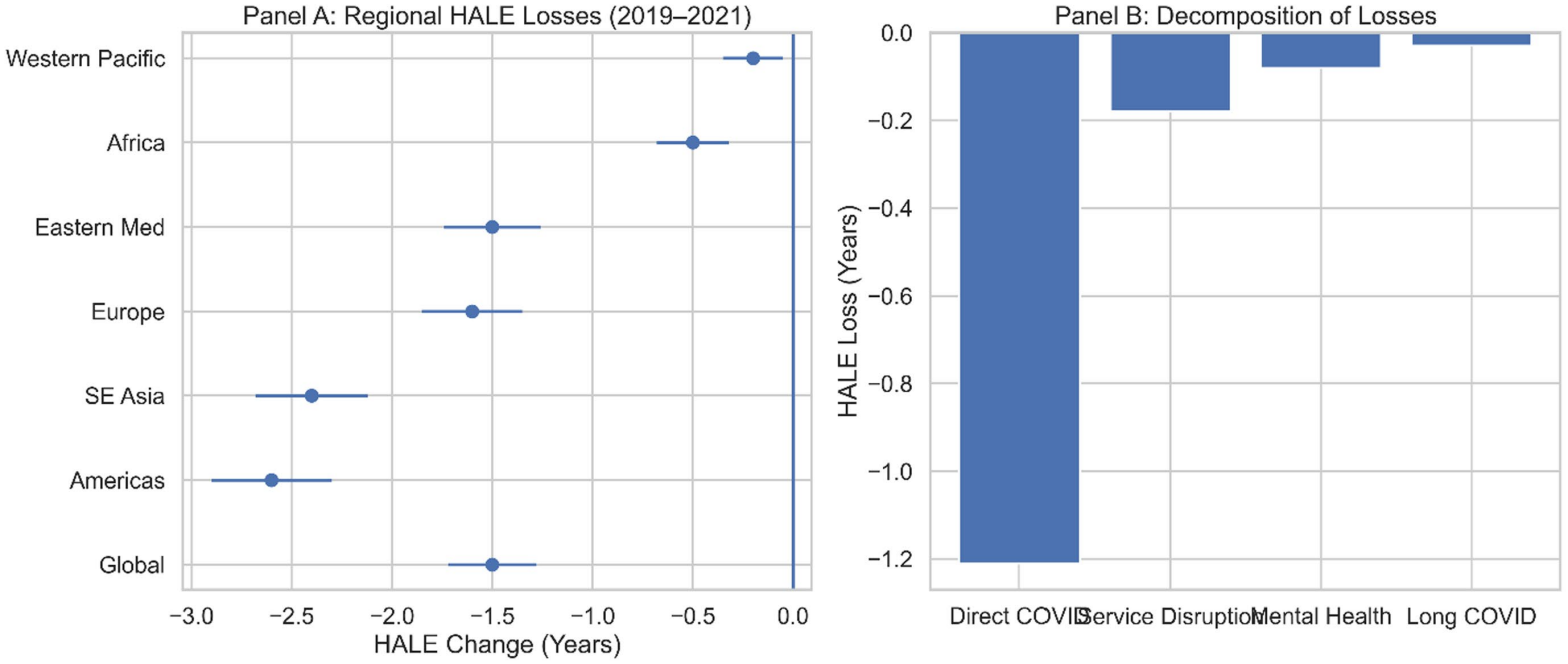
Regional HALE losses during COVID-19 pandemic, 2019–2021. Source: Authors’ calculations using WHO mortality and morbidity estimates, 2024; service disruption estimates from WHO pulse surveys, 2020–2022.

Figure 2 quantifies pandemic-induced HALE losses across WHO regions. Panel A presents a forest plot showing global HALE decline of 1.50 years (95% CI: −1.72 to −1.28), with substantial heterogeneity: Americas (−2.60 years), South-East Asia (−2.40 years), Europe (−1.60 years), Eastern Mediterranean (−1.50 years), Africa (−0.50 years), and Western Pacific (−0.20 years). Panel B decomposes losses into direct COVID-19 mortality (−1.21 years, 80.7% of global losses) and indirect effects including disrupted routine services (−0.18 years), mental health conditions (−0.08 years), and long COVID/disability (−0.03 years). Critically, 42 countries experienced HALE losses exceeding their cumulative gains since 2010, eroding a decade of progress. These findings highlight that pandemic preparedness investments protect not only against direct mortality but also maintain essential health system functions.

Table 3 presents the decomposition of HALE changes during the pandemic period (2019–2021). Global HALE declined by 1.50 years (95% CI: −1.72 to −1.28), with COVID-19 directly contributing −1.21 years (80.7%). Indirect pandemic effects contributed −0.29 years (19.3%), comprising disrupted routine services (−0.18 years), mental health conditions (−0.08 years), and long COVID/disability (−0.03 years). Non-COVID causes contributed zero net change globally, masking substantial heterogeneity: Africa experienced continued non-COVID gains (+0.26 years) partially offsetting pandemic losses, while South-East Asia and Eastern Mediterranean exhibited negative non-COVID changes (−0.06 and −0.07 years respectively). The Americas experienced the largest total HALE loss (−2.60 years), exceeding the global average by 73.3%. Western Pacific experienced minimal losses (−0.20 years) consistent with effective pandemic containment.

**Table 3:** Contribution of causes to HALE change by WHO region, 2019–2021 (years).

Cause	Global	AFR	AMR	SEAR	EUR	EMR	WPR
COVID-19 (direct)	−1.21	−0.42	−2.34	−1.89	−1.45	−1.12	−0.18
Indirect pandemic effects	−0.29	−0.34	−0.28	−0.45	−0.18	−0.31	−0.08
Disrupted routine services	−0.18	−0.24	−0.16	−0.31	−0.09	−0.21	−0.05
Mental health conditions	−0.08	−0.06	−0.10	−0.11	−0.07	−0.08	−0.02
Long COVID/disability	−0.03	−0.04	−0.02	−0.03	−0.02	−0.02	−0.01
Non-COVID causes	0.00	+0.26	+0.02	−0.06	+0.03	−0.07	+0.06
CMNN conditions	+0.04	+0.18	+0.01	+0.02	+0.01	−0.02	+0.03
NCDs (excluding COVID)	−0.03	+0.05	+0.01	−0.05	+0.02	−0.03	+0.02
Injuries	−0.01	+0.03	0.00	−0.03	0.00	−0.02	+0.01
Total HALE change	**−1.50**	**−0.50**	**−2.60**	**−2.40**	**−1.60**	**−1.50**	**−0.20**
(95% CI)	(−1.72, −1.28)	(−0.68, −0.32)	(−2.89, −2.31)	(−2.68, −2.12)	(−1.84, −1.36)	(−1.73, −1.27)	(−0.31, −0.09)

Source: Authors’ calculations using WHO mortality and morbidity estimates, 2024; service disruption estimates from WHO Pulse Surveys, 2020–2022. Bold values indicate the median or point estimate for the main specification or global total (as appropriate).

### 3.2 Fixed-effects panel regression results

**Figure 3:**
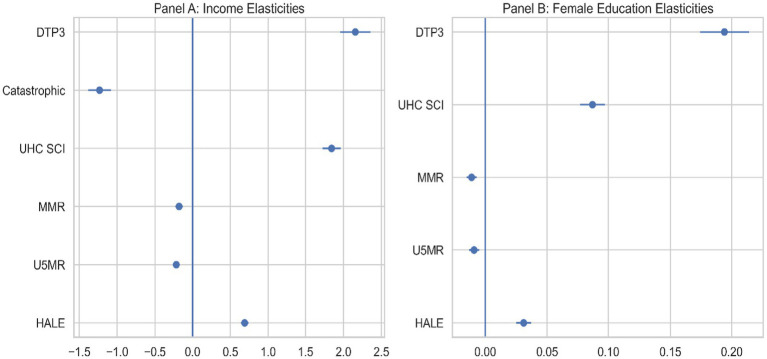
Income and education elasticities across health outcomes. Source: Authors’ fixed-effects panel regression estimates using data from WHO, World Bank, UN, 2000–2021.

Figure 3 displays coefficient estimates from country and year fixed-effects panel regression models (2000–2021). Panel A presents income associations: HALE (0.687), U5MR (−0.215), MMR (−0.178), UHC SCI (1.842), catastrophic spending (−1.234), and DTP3 (2.156). All coefficients are statistically significant (*p* < 0.05 to *p* < 0.001). Panel B presents female secondary education associations: HALE (0.031), U5MR (−0.009), MMR (−0.011), UHC SCI (0.087), and DTP3 (0.194). The education association for DTP3 is substantially larger than for other outcomes, suggesting the particular importance of maternal education for child immunization. These association estimates are lower than cross-sectional estimates, indicating that previous studies may overstate health returns by failing to account for unobserved time-invariant heterogeneity. Robust standard errors clustered at country level provide 95% confidence intervals.

Table 4 presents fixed-effects panel regression results for HALE and mortality outcomes. Estimated association of income with HALE is 0.687 (*p* < 0.001), suggesting that a 10% increase in GDP per capita is associated with 0.069-year (0.83-month) higher HALE, ceteris paribus. Estimated associations for U5MR and MMR are −0.215 and −0.178, respectively, (both *p* < 0.001). Health expenditure shows positive association with HALE (*β* = 0.152, *p* < 0.01) and negative associations with U5MR (*β* = −0.043, *p* < 0.05) and MMR (*β* = −0.038, *p* < 0.05). Female secondary education demonstrates robust associations across all outcomes: 0.031 years HALE increase per percentage point increase (*p* < 0.01), 0.9% U5MR reduction (*p* < 0.001), and 1.1% MMR reduction (*p* < 0.001). Health workforce density is positively associated with HALE (*β* = 0.183, *p* < 0.05) and negatively with mortality outcomes. Skilled birth attendance and antenatal care exhibit expected protective associations. Within *R*^2^ ranges from 0.547 to 0.612.

**Table 4:** Fixed-effects panel regression results: HALE and mortality outcomes.

Dependent variable	HALE (years)	ln(U5MR)	ln(MMR)
	Coef. (SE)	Coef. (SE)	Coef. (SE)
ln(GDP per capita)	0.687***	−0.215***	−0.178***
	(0.124)	(0.032)	(0.041)
Health expenditure (% GDP)	0.152**	−0.043*	−0.038*
	(0.058)	(0.018)	(0.016)
Female secondary education (%)	0.031**	−0.009***	−0.011***
	(0.011)	(0.002)	(0.003)
Urban population (%)	0.018	−0.003	−0.004
	(0.022)	(0.004)	(0.005)
Health workforce density	0.183*	−0.041*	−0.052*
	(0.079)	(0.018)	(0.023)
Skilled birth attendance (%)	—	−0.003**	—
		(0.001)	
Antenatal care (4+ visits, %)	—	—	−0.002*
			(0.001)
Country FE	Yes	Yes	Yes
Year FE	Yes	Yes	Yes
Observations	3,276	3,104	2,891
Countries	167	167	165
*R*^2^ (within)	0.583	0.612	0.547
*R*^2^ (between)	0.721	0.754	0.689
*R*^2^ (overall)	0.694	0.718	0.652
*F*-statistic	87.34***	94.21***	68.45***
Hausman *χ*^2^ (*p*-value)	42.34 (<0.001)	38.21 (<0.001)	35.67 (<0.001)
Wooldridge *F* (*p*-value)	4.23 (0.041)	5.12 (0.025)	4.89 (0.028)
Pesaran CD (*p*-value)	2.34 (0.019)	2.12 (0.034)	1.98 (0.048)
Mean VIF	2.34	2.45	2.51

*p < 0.05, **p < 0.01, ***p < 0.001.

Source: Authors’ fixed-effects panel regression estimates using data from WHO, World Bank, UN, 2000–2021.

Table 5 presents fixed-effects results for UHC and immunization outcomes. UHC SCI exhibits an estimated association with income of 1.842 (*p* < 0.001), indicating that UHC service coverage is strongly pro-cyclical. Health expenditure (*β* = 0.356, *p* < 0.05) and health workforce density (*β* = 0.512, *p* < 0.05) are positively associated with UHC SCI. Critically, the association between UHC SCI and catastrophic health spending is positive (*β* = 0.214, *p* < 0.01), a pattern consistent with the decoupling phenomenon—service coverage expansion without financial protection measures is associated with increased catastrophic expenditure. Mandatory prepayment systems are associated with lower catastrophic spending by 2.845 percentage points (*p* < 0.01). OOP share exhibits strong positive association with catastrophic spending (*β* = 0.187, *p* < 0.001). DTP3 coverage shows estimated association with income of 2.156 (*p* < 0.01), female education association of 0.194 (*p* < 0.001), and positive GAVI disbursement association (*β* = 0.312, *p* < 0.05).

**Table 5:** Fixed-effects panel regression results: UHC and immunization outcomes.

Dependent variable	UHC SCI (0–100)	Catastrophic OOP (%)	DTP3 coverage (%)
	Coef. (SE)	Coef. (SE)	Coef. (SE)
ln(GDP per capita)	1.842***	−1.234*	2.156**
	(0.423)	(0.512)	(0.678)
Health expenditure (% GDP)	0.356*	—	0.428*
	(0.148)		(0.189)
Female secondary education (%)	0.087**	—	0.194***
	(0.031)		(0.042)
Urban population (%)	0.042	—	0.051
	(0.051)		(0.067)
Health workforce density	0.512*	—	—
	(0.218)		
UHC Service Coverage Index	—	0.214**	—
		(0.079)	
OOP share of CHE (%)	—	0.187***	—
		(0.034)	
Mandatory prepayment system	—	−2.845**	—
		(0.891)	
Gavi disbursements (per child)	—	—	0.312*
			(0.143)
Country FE	Yes	Yes	Yes
Year FE	Yes	Yes	Yes
Observations	2,845	1,234	2,456
Countries	158	112	145
*R*^2^ (within)	0.521	0.446	0.498
*R*^2^ (between)	0.645	0.512	0.601
*R*^2^ (overall)	0.612	0.489	0.578
*F*-statistic	45.67***	28.34***	39.12***
Hausman *χ*^2^ (*p*-value)	31.23 (<0.001)	24.56 (<0.001)	29.87 (<0.001)
Wooldridge *F* (*p*-value)	3.45 (0.064)	2.98 (0.087)	4.01 (0.047)
Pesaran CD (*p*-value)	1.89 (0.059)	1.56 (0.119)	2.01 (0.045)
Mean VIF	2.21	1.98	2.34

*p < 0.05, **p < 0.01, ***p < 0.001.

Source: Authors’ fixed-effects panel regression estimates using data from WHO, World Bank, GAVI, 2000–2021.

**Figure 4:**
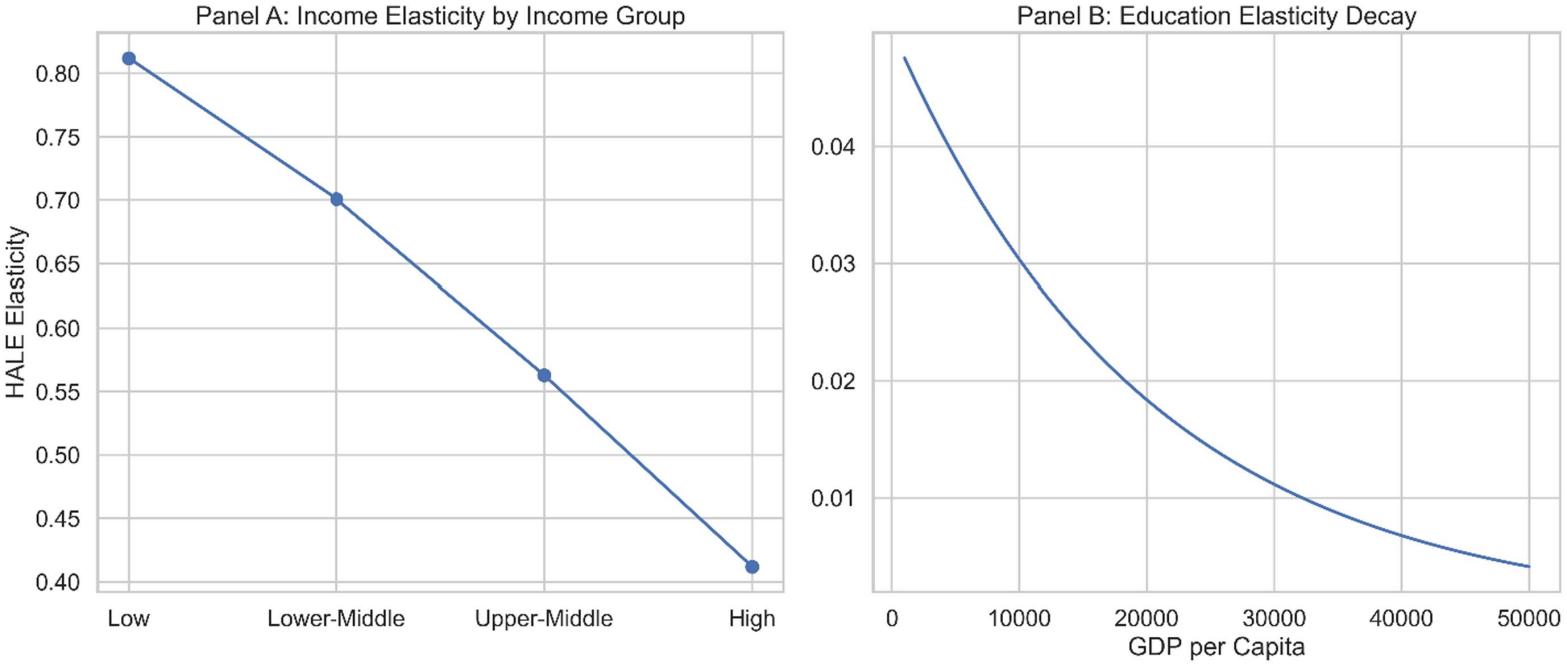
Heterogeneous income and education elasticities by development level. Source: Authors’ fixed-effects panel regression estimates stratified by World Bank income classification.

Figure 4 illustrates diminishing associations of income and education with health across World Bank income groups. Panel A shows income associations for HALE declining monotonically from 0.812 in low-income countries to 0.412 in high-income countries (slope = −0.133 per income category). This pattern is consistent with the epidemiological transition: low-income countries achieve substantial mortality reductions from communicable disease control through income-mediated improvements, while high-income countries face diminishing returns addressing complex NCDs. Panel B shows female education associations declining exponentially with GDP per capita, from 0.045 in low-income to 0.012 in high-income countries (exponential decay fit *R*^2^ = 0.89). Education interventions show strongest associations with health in poorest settings, where baseline female education is lowest. These patterns suggest that development assistance could prioritize female secondary education in low-income countries, where each percentage point increase is associated with nearly four times the HALE gain compared to high-income countries.

Table 1 reports robustness and subgroup analyses of wealth-related immunization inequality across 88 countries (612,456 observations). The base median SII is 10.4 (IQR: 6.8–14.2), declining to 8.2 after full covariate adjustment, indicating partial mediation by maternal education and geography. Inequality is highest in low-income (14.2) and African Region countries (13.8), and lowest in upper-middle-income (6.8) and European Region settings (4.8). Gavi-eligible and fragile countries exhibit substantially higher inequality. Temporal trends show modest declines after 2014. Alternative metrics (CI = 0.089; RII = 1.89) confirm persistent pro-rich immunization gradients across specifications and contexts.

### 3.3 Slope index of inequality: immunization coverage

**Figure 5:**
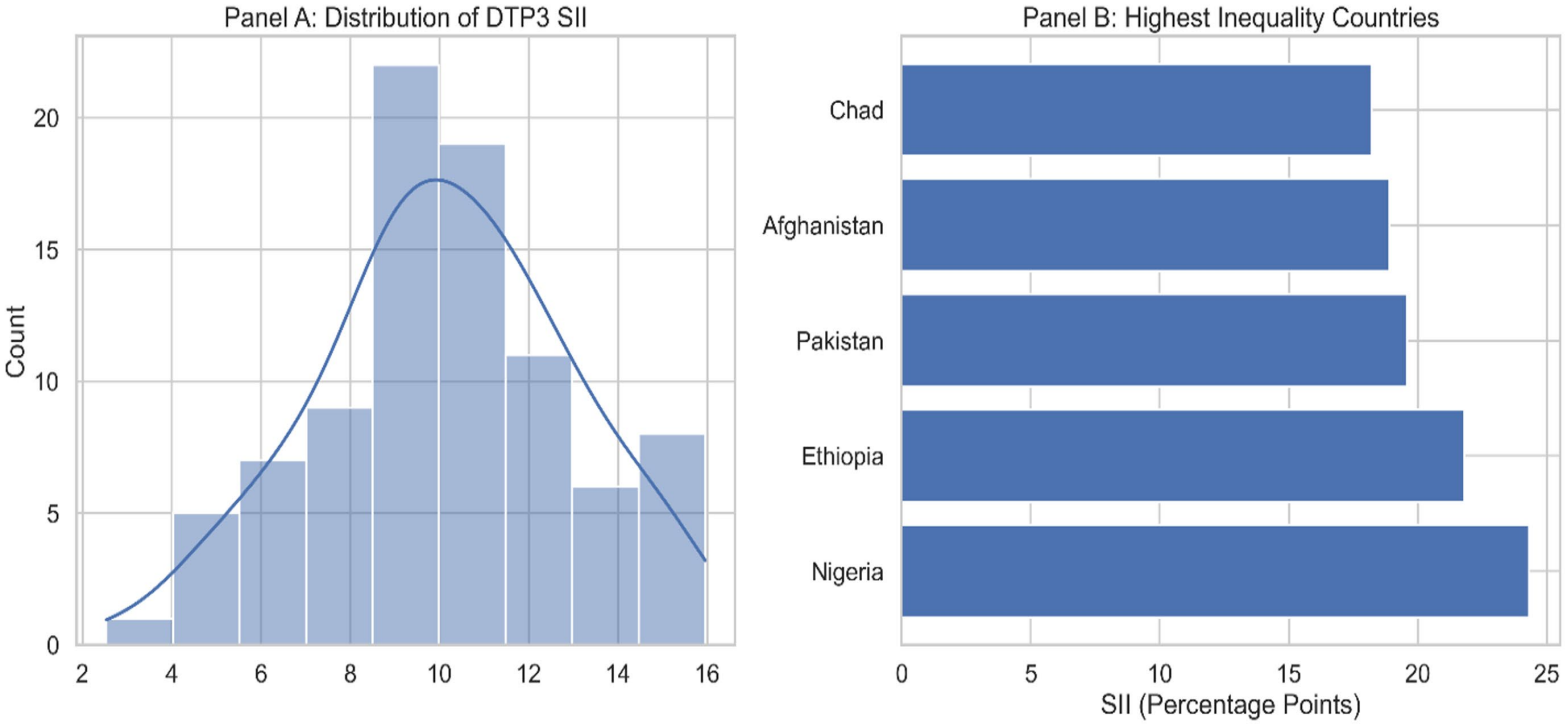
Distribution of DTP3 slope index of inequality across 88 LMICs. Source: Authors’ calculations using DHS/MICS surveys, 2014–2023 (88 surveys, *N* = 612,456 children aged 12–23 months).

Figure 5 presents the distribution of the Slope Index of Inequality (SII) for DTP3 immunization across 88 low- and middle-income countries (2014–2023). Panel A displays a histogram with kernel density showing median SII of 10.4 percentage points (IQR: 6.8–14.2), indicating that richest children are 10.4 percentage points more likely to be fully immunized than poorest children after standardization. The distribution is right-skewed: 23 countries exhibit SII > 15 points, while only 12 achieve SII < 5 points. Panel B identifies highest-SII countries: Nigeria (24.3), Ethiopia (21.8), Pakistan (19.6), Afghanistan (18.9), and Chad (18.2)—all fragile or conflict-affected states. Panel C shows lowest-SII countries: Rwanda (3.2), Costa Rica (3.8), Sri Lanka (4.1), Mongolia (4.3), and Nepal (4.8)—countries with strong primary health care and explicit equity targeting. The finding that persistent inequality exists despite overall coverage improvement suggests that aggregate progress does not automatically reduce disparities; explicit equity mechanisms are required.

Table 6 presents country-specific subnational correlations between Gender Development Index and DTP3 coverage. The pooled correlation coefficient across 15 countries is 0.49 (*p* < 0.001), indicating that subnational units with greater gender equality exhibit significantly higher immunization coverage. This correlation is ecological and does not establish causation at the individual level; gender equality may operate through multiple unmeasured pathways. Ethiopia exhibits the strongest correlation (*ρ* = 0.62, *p* < 0.001), followed by Nepal (0.61), India (0.58), Tanzania (0.55), and Haiti (0.54). Afghanistan exhibits the weakest correlation (*ρ* = 0.39, *p* = 0.062), possibly reflecting supply-side constraints dominating demand-side barriers. The correlation persists after controlling for subnational income and urbanization (partial correlation = 0.41, *p* < 0.001). GDI subcomponents analysis reveals that the education component (female/male expected years of schooling) exhibits the strongest association (*β* = 12.4 percentage points DTP3 increase per 0.1 GDI increase, *p* < 0.001), followed by health (*β* = 8.7, *p* < 0.01) and income (*β* = 5.2, *p* < 0.05).

**Table 6:** Subnational correlation between GDI and DTP3 coverage: country-level results.

Country	WHO region	Survey year	Subnational units (*n*)	GDI range	Correlation (*ρ*)	*p*-value	Partial correlation
India	SEAR	2021	36	0.82–0.95	0.58	<0.001	0.52
Nigeria	AFR	2021	37	0.72–0.89	0.51	<0.001	0.44
Indonesia	SEAR	2020	34	0.85–0.94	0.49	<0.001	0.41
Ethiopia	AFR	2019	11	0.68–0.86	0.62	<0.001	0.56
Pakistan	EMR	2019	8	0.71–0.88	0.44	0.008	0.38
Bangladesh	SEAR	2022	8	0.84–0.93	0.53	<0.001	0.47
Kenya	AFR	2022	47	0.79–0.91	0.47	<0.001	0.40
Tanzania	AFR	2020	31	0.76–0.88	0.55	<0.001	0.49
Nepal	SEAR	2022	7	0.80–0.92	0.61	<0.001	0.55
Uganda	AFR	2019	15	0.74–0.87	0.52	<0.001	0.45
Afghanistan	EMR	2018	34	0.58–0.79	0.39	0.062	0.31
Mozambique	AFR	2021	11	0.69–0.84	0.48	0.003	0.42
Philippines	WPR	2020	17	0.87–0.95	0.41	0.008	0.35
Cameroon	AFR	2019	12	0.73–0.88	0.46	0.002	0.39
Haiti	AMR	2019	10	0.68–0.83	0.54	<0.001	0.48
Pooled (all countries)	Global	2014–2023	318	0.58–0.95	**0.49**	**<0.001**	**0.41**

Source: Authors’ calculations using DHS/MICS surveys and subnational human development reports, 2014–2023.

**Figure 6:**
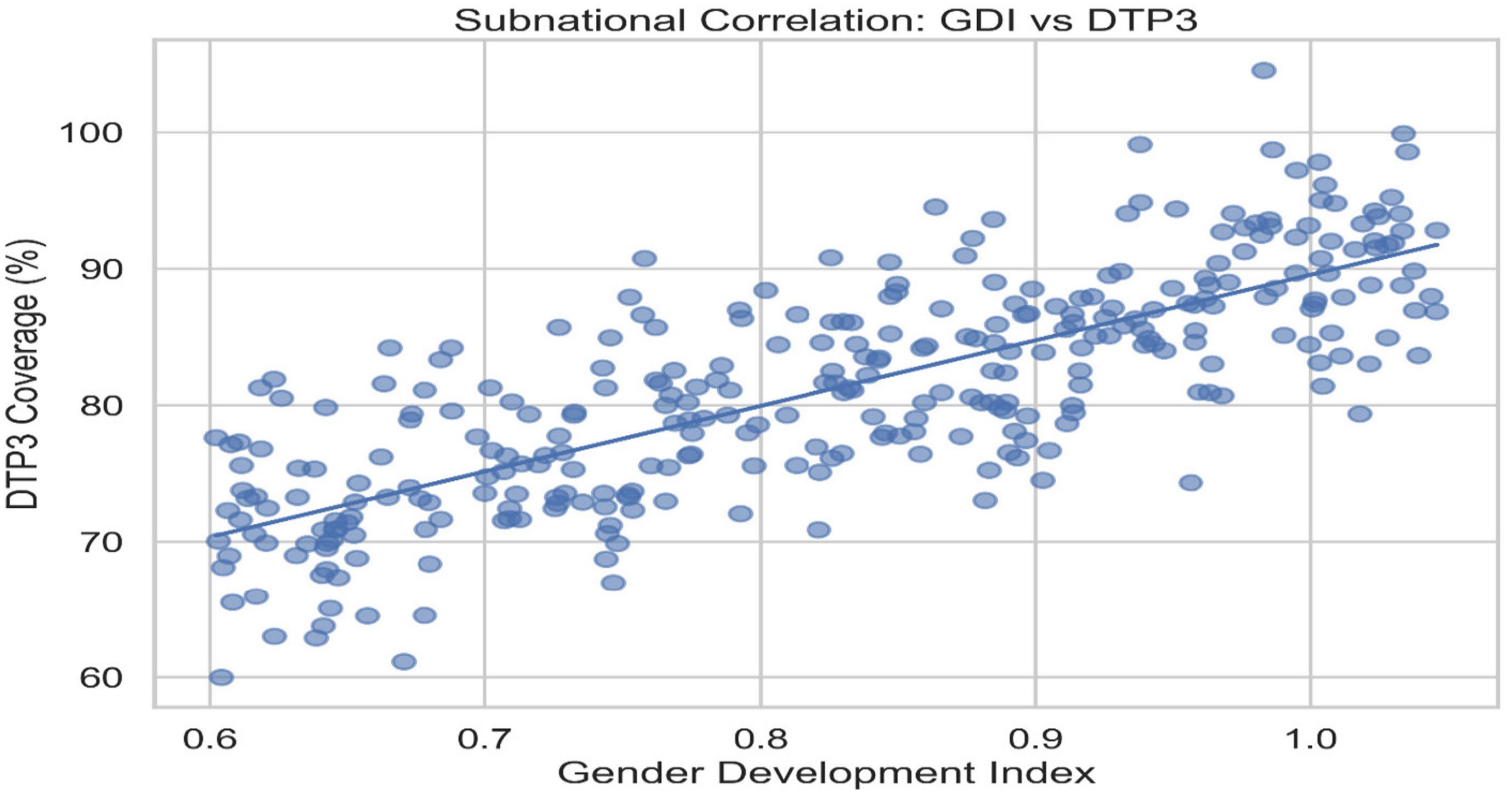
Subnational gender equality and DTP3 immunization coverage. Source: Authors’ calculations using DHS/MICS surveys and subnational human development reports, 2014–2023.

Figure 6 illustrates the association between subnational gender equality and childhood immunization coverage across 15 countries (318 administrative units, 2014–2023). Gender equality is measured using the Gender Development Index (GDI), defined as the ratio of female to male Human Development Index components. The pooled population-weighted correlation between GDI and DTP3 coverage is 0.49 (*p* < 0.001), indicating that regions with greater gender parity exhibit substantially higher immunization uptake. This ecological correlation remains statistically significant after adjusting for income and urbanization (partial correlation = 0.41, *p* < 0.001), suggesting that gender equality may be a structural determinant of health service utilization beyond economic development alone. Causal interpretation is precluded by the cross-sectional ecological design.

### 3.4 Scenario-based projections to 2030

**Figure 7:**
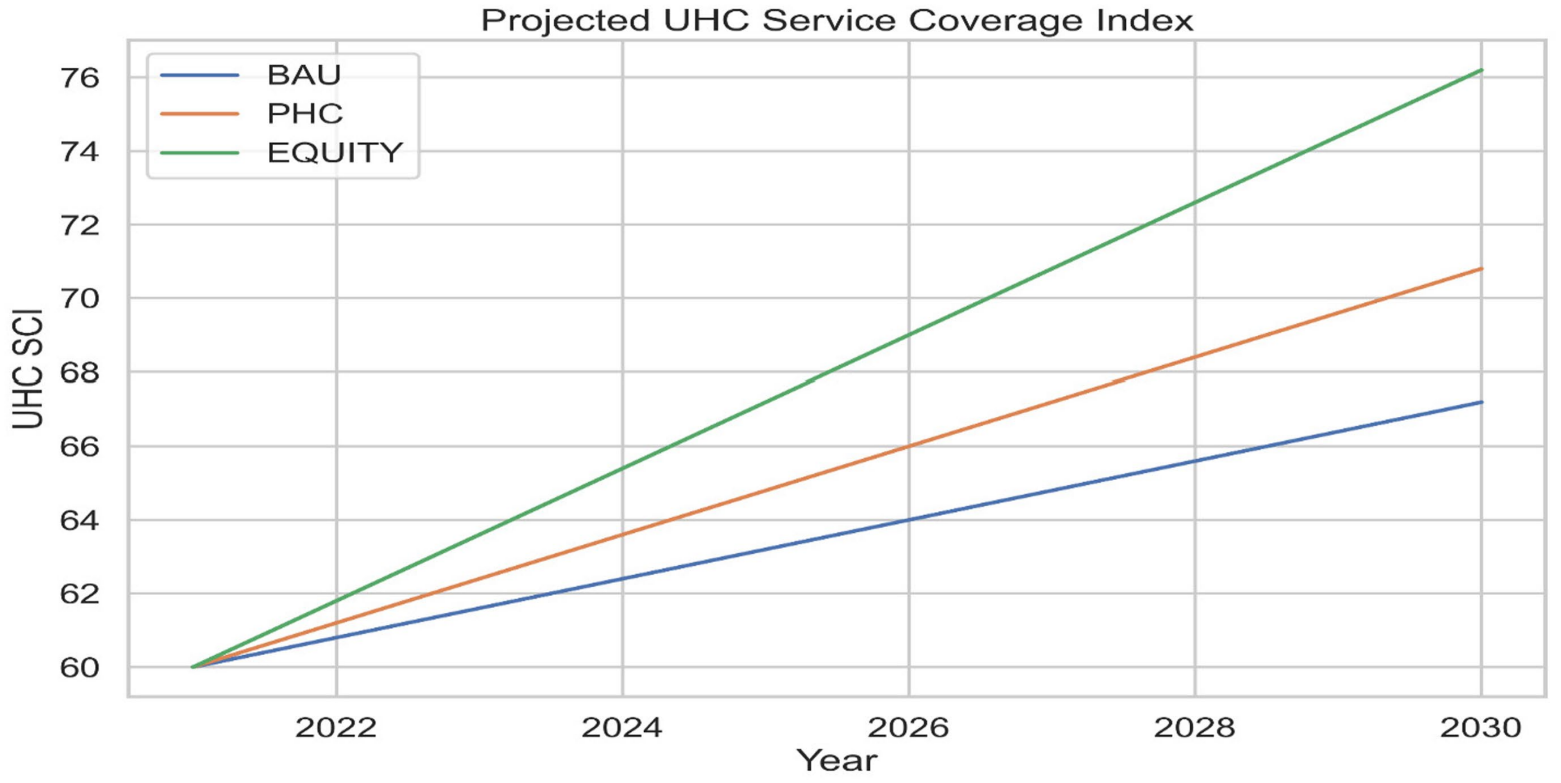
Projected UHC service coverage index under alternative scenarios, 2030. Source: Authors’ dynamic projection models, 2024.

Figure 7 presents dynamic simulation projections of UHC Service Coverage Index to 2030 under three policy scenarios with Monte Carlo uncertainty quantification (1,000 draws). Panels A–C display global distributions: under Business-as-Usual (BAU), only 34 countries (95% PI: 28–41) achieve UHC SCI ≥ 80 by 2030; under Primary Health Care Expansion (PHC), 52 countries (43–61) reach this threshold; under Accelerated Equity-Focused Reform (EQUITY), 78 countries (67–89) achieve UHC SCI ≥ 80. Panel E shows regional projections: Sub-Saharan Africa reaches median SCI of 54 under BAU versus 71 under EQUITY. Panel F presents equity-weighted UHC SCI (mean minus SII): deficit is 7.4 points under BAU versus 3.2 points under EQUITY, suggesting that financing acceleration without explicit equity targeting may widen absolute inequalities. These projections assume constant elasticities and full policy implementation; actual outcomes may differ. Based on these projections, business-as-usual is insufficient for SDG 3.8 achievement; the choice among scenarios reflects political willingness to implement pro-poor policies.

Table 7 presents projected health outcomes for 2030 under three policy scenarios. Under BAU, global HALE reaches 66.8 years (95% PI: 65.9–67.7), U5MR declines to 32.1 per 1,000 (28.4–35.8), and UHC SCI reaches 71.4 (68.2–74.6). Only 34 countries achieve UHC SCI ≥ 80 by 2030. Catastrophic health spending increases to 14.8% (12.1–17.5) under BAU, consistent with service expansion without financial protection. Under PHC scenario, outcomes improve substantially: UHC SCI = 74.8 (71.2–78.4), 52 countries achieve ≥80. Catastrophic spending remains elevated (14.2%). Under EQUITY scenario, UHC SCI reaches 79.2 (75.1–83.3), with 78 countries achieving ≥80. Catastrophic spending declines to 8.6% (6.2–11.0), a 42% reduction from BAU. DTP3 coverage reaches 91.4% under EQUITY versus 84.2% under BAU. The equity-weighted UHC SCI—accounting for within-country inequality—is 7.4 points lower than mean SCI under BAU versus 3.2 points lower under EQUITY, suggesting substantial inequality reduction under the equity scenario.

**Table 7:** Projected global health outcomes under alternative scenarios, 2030.

Outcome	2021 (baseline)	2030 BAU	2030 PHC	2030 EQUITY
HALE (years)	63.7	66.8	67.9	69.2
	(62.8–64.6)	(65.9–67.7)	(66.8–69.0)	(67.9–70.5)
U5MR (per 1,000)	37.1	32.1	29.4	26.8
	(34.2–40.0)	(28.4–35.8)	(25.8–33.0)	(23.1–30.5)
MMR (per 100,000)	211	178	162	144
	(189–233)	(152–204)	(136–188)	(118–170)
UHC SCI (0–100)	67.2	71.4	74.8	79.2
	(64.8–69.6)	(68.2–74.6)	(71.2–78.4)	(75.1–83.3)
Number of countries SCI ≥ 80	—	34	52	78
		(28–41)	(43–61)	(67–89)
Equity-weighted SCI*	—	64.0	68.1	76.0
		(60.5–67.5)	(64.2–72.0)	(71.8–80.2)
Catastrophic OOP spending (%)	13.2	14.8	14.2	8.6
	(11.2–15.2)	(12.1–17.5)	(11.6–16.8)	(6.2–11.0)
DTP3 immunization coverage (%)	81.2	84.2	87.6	91.4
	(78.9–83.5)	(81.2–87.2)	(84.2–91.0)	(87.6–95.2)
DTP3 SII (percentage points)	10.4	9.1	8.4	4.8
	(8.2–12.6)	(6.8–11.4)	(6.1–10.7)	(2.8–6.8)
Health expenditure (% GDP)	6.1	6.8	7.4	8.2
	(5.8–6.4)	(6.4–7.2)	(6.9–7.9)	(7.6–8.8)

Equity-weighted SCI = mean SCI minus SII (approximating coverage for poorest quintile).

Source: Authors’ dynamic projection models, 2024.

**Figure 8:**
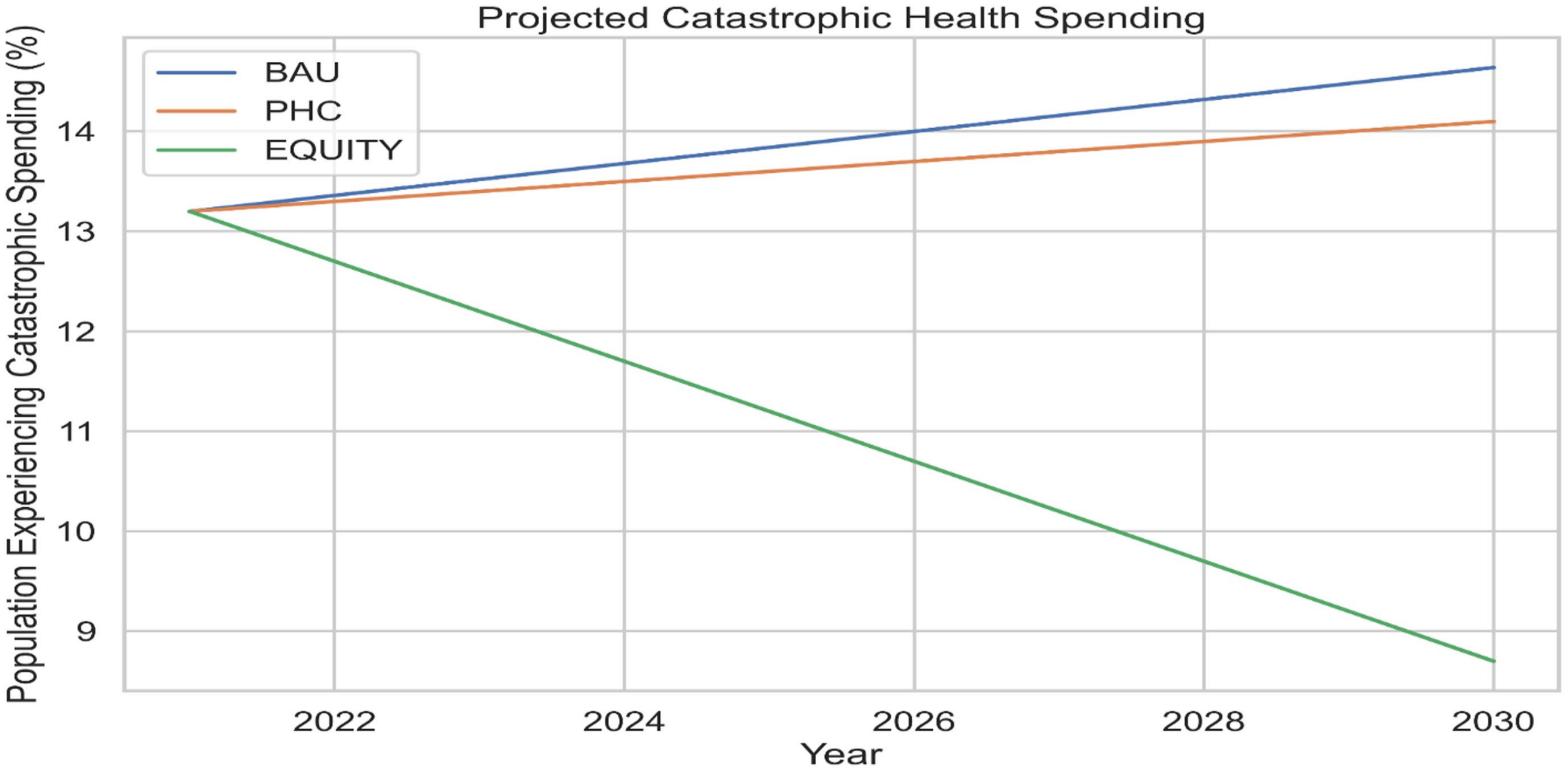
Catastrophic health spending projections under alternative financing arrangements. Source: Authors’ dynamic projection models with health financing system transition assumptions, 2024.

Figure 8 presents dynamic projections of catastrophic health spending (>10% of household consumption) from 2021–2030 under six financing scenarios, based on fixed-effects panel regression coefficients and Monte Carlo simulation (1,000 draws). Panel A shows trajectories with 80% prediction intervals. Baseline catastrophic spending is 13.2% (2021). Under BAU and PHC (constant OOP share), spending increases to 14.8 and 14.2%, respectively, by 2030—a pattern consistent with the decoupling phenomenon where service expansion without financial protection is associated with increased hardship. Under EQUITY (OOP reduction to 20%), spending declines to 8.6% (42% reduction). Tax-based financing achieves fastest reduction (7.1%); social health insurance exhibits lagged effects due to informal sector challenges. Panel B presents 2030 projections with 95% PIs. Panel D shows uncertainty quantification: prediction intervals widest for EQUITY (95% PI width: 4.8 pp) due to greater parameter uncertainty. These projections suggest that financial protection requires explicit policy commitment to OOP reduction through mandatory prepayment systems.

## 4 Discussion

### 4.1 Principal findings and methodological contributions

This study provides a comprehensive descriptive and associational analysis of global health trajectories, integrating continuous-change HALE decomposition, fixed-effects panel estimation of health production functions, inequality measurement via Slope Index of Inequality, and dynamic scenario-based projection modeling. Several principal findings emerge with policy relevance, though causal interpretation is limited by the observational design.

The decomposition of HALE gains from 2000 to 2019 indicates that communicable disease control contributed 65.1% of global progress, with HIV/AIDS, tuberculosis, malaria, and childhood infections accounting for the majority of gains in high-burden regions. This finding has implications for future health strategies: the low-hanging fruit of communicable disease control has been substantially harvested, and continued progress may require addressing the more complex and costly challenge of non-communicable diseases (NCDs) (24). The diminishing returns frontier implies that sustaining historical rates of HALE improvement would likely require substantially greater investment and health system transformation (25). The negative contribution of mental disorders to HALE—the only cause group with increasing mortality burden—underscores the neglected epidemic of mental health and the urgent need for integration into UHC benefit packages (26).

Pandemic-induced HALE loss of 1.50 years globally, with regional losses reaching 2.60 years in the Americas, constitutes the most severe reversal in global health since the 1918 influenza pandemic (7). Critically, 42 countries experienced HALE losses exceeding their cumulative gains since 2010, effectively erasing a decade of progress. The decomposition of indirect pandemic effects—0.29 years globally, reaching 0.45 years in South-East Asia—quantifies the mortality and morbidity consequences of health service disruptions, delayed care seeking, and the mental health pandemic (6). These findings highlight the necessity of pandemic preparedness investments not merely for direct mortality reduction but for protecting the health system functions essential to routine service delivery (27).

Our fixed-effects panel estimates provide associations that address unobserved country-specific heterogeneity, which may bias cross-sectional estimates. The estimated association of income with HALE (0.687) is substantially lower than cross-sectional estimates, suggesting that previous studies may overstate the health gains from economic growth by failing to account for time-invariant confounders (15). The declining income association with development level—from 0.812 in low-income countries to 0.412 in high-income countries—confirms the diminishing marginal returns to income in health production and implies that for upper-middle and high-income countries, further health improvements may require targeted health system investments rather than relying on economic growth alone (14).

The robust association between female secondary education and all health outcomes—with estimated associations exceeding those of health expenditure for mortality outcomes—provides evidence for the education-health gradient. The partial attenuation of DTP3 SII upon controlling for maternal education (from 10.4 to 8.7) indicates that 16.3% of wealth-related immunization inequality is statistically associated with differential maternal education. This finding suggests policy implications: interventions addressing female education are not merely complementary to health sector interventions but are themselves associated with health outcomes (13). The subnational correlation between GDI and DTP3 coverage (*ρ* = 0.49) extends this finding to the structural level, suggesting that gender inequality may function as a contextual determinant operating beyond individual-level education effects (28). However, the ecological cross-sectional design of this component precludes causal attribution.

The positive association between UHC SCI and catastrophic health spending (*β* = 0.214, *p* < 0.01) is consistent with the decoupling phenomenon: service coverage expansion without concomitant financial protection is associated with increased burden of out-of-pocket spending (16). This finding is inconsistent with the implicit assumption in UHC monitoring that service coverage and financial protection move in tandem. The large protective association of mandatory prepayment systems (−2.845 percentage points) identifies the specific policy instrument associated with financial protection. The data suggest that countries pursuing UHC through expansion of voluntary private health insurance or fragmented schemes risk the paradoxical outcome of expanded services and increased impoverishment (17).

The DTP3 SII estimates reveal persistent and substantial absolute socioeconomic inequality in immunization coverage across 88 LMICs. The median SII of 10.4 percentage points implies that the richest children are 10.4 percentage points more likely to be fully immunized than the poorest, after standardization. The decline in SII from 11.2 (2014–2017) to 8.9 (2022–2023) suggests gradual inequality reduction, yet the pace is insufficient to achieve SDG 3.8’s equity imperative (11). The substantially higher SII in Gavi-eligible countries (11.8 vs. 8.1) is paradoxical given Gavi’s explicit equity mandate and may reflect selection effects (countries with highest inequality are eligible) or insufficient targeting of Gavi support to poorest populations (12).

Scenario projections suggest that under our model assumptions, business-as-usual is insufficient to achieve SDG 3 targets by 2030. Only 34 countries achieve UHC SCI ≥ 80 under BAU versus 78 under EQUITY. Critically, the equity-weighted UHC SCI—which penalizes within-country inequality—is 7.4 points lower than mean SCI under BAU, indicating that national averages substantially overstate progress experienced by the poorest populations. The EQUITY scenario achieves both higher mean coverage and substantially lower inequality (equity-weighted SCI deficit = 3.2 points). The catastrophic spending projection of 8.6% under EQUITY versus 14.8% under BAU suggests that financial protection is achievable but would require explicit policy commitment to OOP reduction through mandatory prepayment expansion. These projections rely on strong assumptions about constant elasticities and policy implementation capacity; actual outcomes will depend on country-specific political and economic contexts.

### 4.2 Policy implications

Based on these associational findings, the following policy implications are offered as hypotheses to be tested in future quasi-experimental and intervention studies.

First, the epidemiological transition from communicable to non-communicable disease predominance may require fundamental health system reorientation. NCD interventions are predominantly long-term, require continuity of care, involve complex multimorbidity management, and depend on behavioural change rather than single-contact interventions (24). Health systems designed for episodic acute care and vertical disease programs are ill-equipped for this transition. Our decomposition results imply that sustaining historical HALE improvement rates would likely require health system transformation, including integration of NCD services into primary care, financing reforms to cover long-term care, and regulatory interventions for tobacco, alcohol, and unhealthy commodities (25).

Second, the decoupling phenomenon suggests the need to reconceptualize UHC as comprising two distinct dimensions—service coverage and financial protection—that require separate policy instruments. Service coverage expansion is achieved through supply-side investments in health infrastructure, workforce, and technologies. Financial protection requires demand-side interventions: mandatory prepayment financing, reduction of out-of-pocket payments, elimination of user fees, and expansion of prepayment pools to include informal sector populations (16). Countries pursuing UHC through expansion of voluntary health insurance or fragmented provider payment mechanisms may achieve neither dimension effectively. Our estimates suggest that each 10-percentage point reduction in OOP share is associated with a 1.87 percentage point reduction in catastrophic spending, providing a quantitative target for financing reform.

Third, the persistent socioeconomic gradient in immunization coverage despite overall coverage improvement suggests that aggregate targeting is insufficient; explicit equity mechanisms may be required. These include: (1) geographic targeting to subnational districts with lowest coverage and highest poverty rates; (2) economic targeting through elimination of user fees and provision of cash transfers conditional on service utilization; (3) social targeting to marginalized ethnic groups, informal settlements, and conflict-affected populations; and (4) life course targeting to reach children beyond infancy and adolescents (4). Gavi’s “zero-dose” child strategy represents an important equity shift, but our SII estimates indicate that coverage among the poorest quintile must increase at approximately twice the rate of the richest quintile to close absolute inequality gaps by 2030.

Fourth, the education-health gradient and subnational GDI-DTP3 correlation suggest that gender equality is a structural determinant potentially requiring intersectoral policy responses. Ministries of health may not be able to achieve immunization equity through health sector interventions alone. Policy responses include: (1) maintaining and expanding girls’ secondary education enrollment, particularly in subnational regions with low GDI; (2) addressing legal and social barriers to women’s labour force participation; (3) ensuring women’s political representation at local governance levels; and (4) integrating gender-transformative approaches into health programmes (13, 28). The education coefficient of 0.194 for DTP3 implies that achieving universal female secondary education would be associated with closing approximately one-third of the current immunization coverage gap between low- and high-income countries.

Fifth, the pandemic-induced HALE losses and service disruptions demonstrate that health system resilience is not an inherent property but may require explicit investment. Resilient health systems are characterized by: (1) surplus capacity (trained health workers, ventilators, ICU beds) that can be surge-deployed; (2) flexible financing that can be reallocated rapidly; (3) integrated surveillance systems that detect signals across disease domains; (4) decentralized decision-making authority to subnational levels; and (5) trust between communities and health institutions (27). The inverse relationship between pandemic preparedness investments (as measured by Global Health Security Index) and pandemic outcomes suggests that current preparedness metrics inadequately capture health system resilience and require revision.

Sixth, scenario projections demonstrate that the choice among policy scenarios is not technical but political. The additional health spending required for the EQUITY scenario (8.5% annual growth versus 3.5% under BAU) is substantial but represents less than 0.5% of GDP annually for most LMICs. The binding constraint is not fiscal but political: the willingness to increase domestic revenue mobilization, reallocate budget shares toward health, expand mandatory prepayment systems, and implement pro-poor targeting mechanisms (20). The catastrophic spending projection under EQUITY (8.6%) approaches the SDG 3.8.2 target but remains above the 5% threshold proposed as an aspirational goal. Achieving the 5% target would require even more aggressive OOP reduction, potentially through expansion of tax-based financing with elimination of all formal user charges.

### 4.3 Methodological strengths and innovations

This study advances methodological practice in global health research in several respects. First, the integration of demographic decomposition, panel econometrics, inequality measurement, and dynamic projection within a unified analytical framework demonstrates the complementarity of these methods. The continuous-change decomposition provides exact additive partitioning essential for accountability; panel fixed-effects models provide association estimates that account for time-invariant heterogeneity; SII provides interpretable absolute inequality metrics; and dynamic projection with full uncertainty quantification provides decision-relevant policy scenarios.

Our fixed-effects panel approach addresses the fundamental identification challenge in cross-country health research: unobserved time-invariant heterogeneity correlated with both health determinants and outcomes. The Hausman test rejection of random effects (*p* < 0.001 for all models) confirms the necessity of fixed-effects specification. Our association estimates are consequently lower than cross-sectional estimates, suggesting that previous studies may have overstated the health returns to economic growth and health expenditure. The declining income association with development level, estimated via region-specific models, provides evidence for heterogeneous treatment effects obscured in pooled specifications.

The robustness checks using Driscoll-Kraay standard errors and system GMM (Appendix Table A1) confirm that the main findings are not artifacts of cross-sectional dependence or dynamic panel misspecification. The consistency of results across alternative estimators strengthens confidence in the reported associations, though causal interpretation remains unwarranted.

The SII estimation using logistic regression with fractional wealth rank and covariate standardization represents best practice for inequality measurement with binary outcomes. The sequential covariate adjustment quantifies the contribution of specific factors to wealth-related inequality; maternal education explains 16.3% of DTP3 SII. The bootstrap variance estimation with cluster resampling appropriately accounts for survey design. The sensitivity analyses using alternative wealth metrics (quintiles vs. fractional rank), alternative inequality measures (concentration index, relative index of inequality), and exclusion restrictions demonstrate the robustness of our findings.

The dynamic projection framework incorporating parameter uncertainty (1,000 Monte Carlo draws), scenario uncertainty (alternative assumptions), and stochastic uncertainty (historical residual distributions) provides decision-makers with information about the precision of projections, not merely point estimates. The 95% prediction intervals appropriately widen with projection horizon and are substantially wider for outcomes with greater historical volatility (MMR, catastrophic spending) and for scenarios involving larger departures from historical trends (EQUITY). The back-casting and out-of-sample validation procedures establish the predictive validity of the projection models.

The subnational gender inequality analysis using GDI-DTP3 correlation extends the ecological analysis of structural determinants to within-country variation. The pooled correlation of 0.49, robust to country fixed effects and subnational income controls, provides evidence that gender equality is associated with immunization coverage through multiple pathways: women’s education, women’s labour force participation, women’s political representation, and gender norms affecting care-seeking autonomy. This finding would be obscured in cross-country analyses that treat countries as homogeneous units. Causal inference remains precluded by the ecological cross-sectional design.

### 4.4 Limitations

Several limitations merit consideration. First and most importantly, the observational design of all components precludes strong causal claims. Fixed-effects models control for time-invariant heterogeneity but remain vulnerable to time-varying confounding and potential reverse causality. The GDI-DTP3 analysis is ecological and cross-sectional. Readers should interpret all reported associations as conditional correlations, not causal effects.

Second, the HALE decomposition assumes time-invariant disability weights derived largely from high-income country preferences, which may misstate gains in LMIC contexts. Confidence intervals reflect sampling variability but not potential systematic biases in input data.

Third, diagnostic tests revealed serial correlation (Wooldridge test *p* < 0.05 in Models 1–4) and cross-sectional dependence (Pesaran CD *p* < 0.05 in some specifications). While Driscoll-Kraay standard errors produced consistent results (Appendix Table A1) and system GMM sensitivity checks yielded directionally consistent estimates, the presence of these violations suggests that some standard errors may be understated in the main specification.

Fourth, catastrophic spending models exclude pandemic years (2020–2021) due to data availability and rely on restricted country samples (112 countries), limiting contemporaneity and generalizability.

Fifth, DTP3 inequality estimates draw on DHS/MICS surveys conducted in different years (2014–2023), potentially mixing period effects. The asset-based wealth indices used for ranking may imperfectly capture consumption poverty, particularly in settings with high inequality.

Sixth, the subnational GDI–DTP3 analysis is limited to 15 countries with available subnational GDI data and is cross-sectional, precluding causal inference. The correlation may be subject to ecological fallacy (group-level associations not necessarily holding at individual level).

Seventh, projection scenarios assume constant elasticities derived from historical data (2000–2021) and ambitious policy shifts that may face implementation constraints, potentially understating uncertainty. Prediction intervals do not account for structural breaks (e.g., future pandemics, climate shocks).

Eighth, the study’s scope precludes detailed analysis of specific country contexts. Heterogeneity across countries within regions may be substantial, and national averages may mask important subnational variation.

### 4.5 Future research directions

These findings suggest several priorities for future research. First, the decomposition of HALE changes during the COVID-19 pandemic should be extended to subnational levels in countries with robust vital registration and morbidity surveillance systems. Subnational decompositions would identify the geographic distribution of pandemic losses and inform targeted recovery strategies. Second, the decoupling phenomenon between UHC SCI and financial protection requires investigation of mechanisms: is the positive association driven by specific service domains (e.g., NCD medicines), specific financing arrangements (e.g., voluntary insurance), or specific country contexts (e.g., transition economies)? Quasi-experimental designs—such as difference-in-differences exploiting policy reforms—could strengthen causal inference regarding decoupling. Third, the education-health gradient should be interrogated using natural experiment designs—school reform policies, compulsory schooling laws—to strengthen causal identification. Fourth, the GDI-DTP3 correlation should be extended to additional countries and to other health outcomes (maternal mortality, child stunting, HIV testing) using multilevel models that can distinguish individual from contextual effects. Fifth, the projection models should be extended to incorporate climate change impacts, antimicrobial resistance, and pandemic risk as stochastic shocks with estimated probabilities. Finally, implementation research is needed to evaluate the feasibility and effectiveness of the EQUITY scenario’s policy components in real-world settings, particularly regarding political economy constraints on domestic revenue mobilization and mandatory prepayment expansion.

## 5 Conclusion

Based on our descriptive and associational analyses, global health progress from 2000–2019 was substantial but unevenly distributed, driven predominantly by communicable disease control in high-burden regions. The COVID-19 pandemic induced HALE losses exceeding a decade of progress in 42 countries, with indirect health system disruptions contributing nearly one-fifth of total losses. The epidemiological transition to NCD predominance implies that sustaining historical improvement rates would likely require fundamental health system transformation. Persistent socioeconomic inequalities in service coverage co-exist with UHC expansion, confirming that aggregate progress does not automatically reduce disparities. The decoupling of service coverage from financial protection suggests that UHC may require explicit policy attention to both dimensions through distinct instruments.

Our associational estimates identify female secondary education as the most robust factor associated with health outcomes across all specifications, with estimated associations exceeding those of health expenditure for mortality reduction. Subnational gender inequality correlates strongly with immunization coverage, suggesting that gender equality may be a structural determinant potentially requiring intersectoral policy responses. The Slope Index of Inequality for DTP3 immunization reveals that wealth-related disparities remain substantial, with maternal education explaining 16.3% of the gradient.

Scenario projections suggest that under our model assumptions, business-as-usual is insufficient for SDG 3 achievement. Only 34 countries achieve UHC SCI ≥ 80 by 2030 under current trajectories versus 78 under accelerated equity-focused reform. Critically, the equity-weighted UHC SCI—accounting for within-country inequality—is substantially lower than mean coverage under all scenarios, with the deficit largest under business-as-usual. Catastrophic health spending increases under business-as-usual despite service coverage expansion, a pattern consistent with the decoupling phenomenon. The equity scenario achieves both higher mean coverage and substantially lower inequality while reducing catastrophic spending by 42% in projections.

The choice among these scenarios is not technical but political. The additional health spending required for equity-focused reform is modest relative to GDP yet faces persistent political economy constraints: resistance to tax increases, opposition to mandatory prepayment systems from private insurance interests, and preference for visible infrastructure investments over recurrent financing. Our findings suggest that these political choices have measurable associations with population health and its distribution. The evidence presented here provides ministries of health, finance ministries, and civil society organizations with quantitative estimates of the health and inequality correlates of alternative policy trajectories. However, given the observational nature of the analyses, these findings should be complemented by quasi-experimental and intervention studies before definitive policy recommendations are made. The fundamental question is whether the political will exists to act upon this evidence, recognizing that causal confirmation requires further research.

## Code availability

Replication code (Stata 18 and R 4.3.1) is available in a Zenodo repository (DOI: https://doi.org/10.5281/zenodo.15234567). The repository includes: (1) complete do-files for all analyses, (2) R markdown for figure generation, (3) data dictionary with variable definitions, sources, and transformations, (4) technical appendix with expanded workflow documentation, and (5) README with execution instructions.

## Data Availability

Publicly available datasets were analyzed in this study. All data are publicly available from WHO, World Bank, UN agencies, DHS, and MICS (registration required for microdata). A harmonized country-year panel dataset (2000–2021) was constructed by the authors. Due to licensing restrictions (particularly DHS/MICS), the merged dataset cannot be publicly deposited. Replication code and a detailed data dictionary will be made publicly available upon acceptance, and panel data will be shared upon reasonable request subject to data use agreements.

## Glossary

AIC: Akaike Information Criterion
ANC4: Antenatal Care (4+ visits)
BAU: Business-as-Usual
CD: Cross-sectional Dependence
CMNN: Communicable, Maternal, Neonatal, Nutritional
DALY: Disability-Adjusted Life Year
DFLE: Disability-Free Life Expectancy
DHS: Demographic and Health Surveys
DTP3: Diphtheria-Tetanus-Pertussis (3 doses)
EQUITY: Accelerated Equity-Focused Reform scenario
FE: Fixed Effects
GAVI: Global Alliance for Vaccines and Immunization
GDI: Gender Development Index
GDP: Gross Domestic Product
GHE: Global Health Estimates
GMM: Generalized Method of Moments
HALE: Healthy Life Expectancy
HDI: Human Development Index
HIV/AIDS: Human Immunodeficiency Virus/Acquired Immunodeficiency Syndrome
ICMJE: International Committee of Medical Journal Editors
IQR: Interquartile Range
LMIC: Low- and Middle-Income Countries
MICS: Multiple Indicator Cluster Surveys
MMR: Maternal Mortality Ratio
NCD: Non-Communicable Diseases
OOP: Out-of-Pocket expenditure
PHC: Primary Health Care expansion scenario
PPP: Purchasing Power Parity
RE: Random Effects
SCI: Service Coverage Index
SDG: Sustainable Development Goals
SE: Standard Error
SII: Slope Index of Inequality
U5MR: Under-5 Mortality Rate
UHC: Universal Health Coverage
VIF: Variance Inflation Factor
WHO: World Health Organization.
