# Supplementary material for: Global health trajectories: analysis of health production functions and inequality decomposition
Source: Front Public Health. 2026 May 25;14:1811111. doi: 10.3389/fpubh.2026.1811111 (PMC13243399; doi:10.3389/fpubh.2026.1811111)
Supplement: Supplementary file 1 [file Supplementary_file_1.DOCX]

**Appendix Table A1: Robustness Checks – Comparison of Clustered SE, Driscoll‑Kraay SE, and System GMM Estimates**

| Outcome & Predictor | FE Coefficient (Clustered SE) | Driscoll‑Kraay SE | % Change in SE | System GMM Coefficient (SE) | 95% CI (GMM) | Hansen J p-value | AR (2) p-value |
| --- | --- | --- | --- | --- | --- | --- | --- |
| HALE (years) | | | | |  |  |  |
| ln(GDP per capita) | 0.687*** (0.124) | 0.142 | +14.5% | 0.621* (0.187) | (0.254, 0.988) | 0.213 | 0.184 |
| Health expenditure (% GDP) | 0.152** (0.058) | 0.067 | +15.5% | 0.138* (0.071) | (-0.001, 0.277) | 0.287 | 0.201 |
| Female secondary education (%) | 0.031** (0.011) | 0.013 | +18.2% | 0.027* (0.014) | (-0.001, 0.055) | 0.341 | 0.176 |
| UHC SCI (0‑100) | |  |  |  |  |  |  |
| ln(GDP per capita) | 1.842*** (0.423) | 0.489 | +15.6% | 1.698** (0.612) | (0.498, 2.898) | 0.198 | 0.223 |
| Health expenditure (% GDP) | 0.356* (0.148) | 0.172 | +16.2% | 0.321* (0.189) | (-0.049, 0.691) | 0.265 | 0.198 |
| ln(U5MR) |  |  |  |  |  |  |  |
| ln(GDP per capita) | -0.215*** (0.032) | 0.037 | +15.6% | -0.198*** (0.048) | (-0.292, -0.104) | 0.178 | 0.312 |
| Female secondary education (%) | -0.009*** (0.002) | 0.0025 | +25.0% | -0.008*** (0.003) | (-0.014, -0.002) | 0.245 | 0.287 |
| DTP3 coverage (%) | | | | | | | |
| ln(GDP per capita) | 2.156** (0.678) | 0.789 | +16.4% | 1.987* (0.921) | (0.182, 3.792) | 0.312 | 0.198 |
| Female secondary education (%) | 0.194*** (0.042) | 0.049 | +16.7% | 0.178*** (0.058) | (0.064, 0.292) | 0.287 | 0.234 |

**NOTES:**

- FE = fixed effects with country and year dummies; standard errors clustered at country level.
- Driscoll‑Kraay standard errors account for cross‑sectional dependence and heteroskedasticity; % change in SE is relative to clustered SE.
- System GMM (Arellano‑Bond) uses collapsed instruments (one lag per variable) with robust SE; Hansen J tests overidentifying restrictions (null: instruments valid); AR(2) tests second‑order serial correlation (null: no AR(2)).
- *p<0.05, **p<0.01, ***p<0.001.
- Full results for all outcomes and additional diagnostic statistics are available in the online replication repository (Zenodo: <https://doi.org/10.5281/zenodo.xxxxx>).

**Appendix B: Expanded Replication Workflow Documentation**

This appendix provides detailed documentation of the data processing, interpolation rules, and harmonization procedures used to construct the analytic dataset for the main manuscript. All steps are implemented in the replication code (Stata 18 and R 4.3.1) available in the online repository.

**B.1 Data Sources and Acquisition**

| Variable | Source | Original Frequency | Access Date |
| --- | --- | --- | --- |
| HALE | WHO Global Health Estimates 2024 | Annual (2000‑2021) | 15 March 2024 |
| U5MR | UN IGME | Annual (2000‑2021) | 15 March 2024 |
| MMR | WHO/UNICEF | Annual (2000‑2021) | 15 March 2024 |
| UHC SCI | WHO Global Health Observatory | Annual (2005‑2021) | 20 March 2024 |
| Catastrophic spending | WHO/World Bank | Biennial (2005‑2019) | 20 March 2024 |
| DTP3 coverage | WHO/UNICEF WUENIC | Annual (2000‑2021) | 22 March 2024 |
| GDP per capita (PPP) | World Bank WDI | Annual (2000‑2021) | 25 March 2024 |
| Health expenditure (% GDP) | WHO GHED | Annual (2000‑2021) | 25 March 2024 |
| Female secondary education (%) | UNESCO UIS | Annual (2000‑2021) | 28 March 2024 |
| Urban population (%) | UN World Urbanization Prospects | Annual (2000‑2021) | 28 March 2024 |
| Health workforce density | WHO Global Health Observatory | Annual (2000‑2021) | 30 March 2024 |
| Skilled birth attendance (%) | WHO/UNICEF | Annual (interpolated) | 30 March 2024 |
| Antenatal care (4+ visits, %) | WHO/UNICEF | Annual (interpolated) | 30 March 2024 |
| OOP share of CHE (%) | WHO GHED | Annual (2000‑2021) | 1 April 2024 |
| Mandatory prepayment system | WHO Health Financing Database | Year of reform | 1 April 2024 |
| GAVI disbursements (per child) | GAVI CSO Portal | Annual (2000‑2021) | 3 April 2024 |
| DHS/MICS microdata | DHS Program / UNICEF MICS | Survey rounds (2014‑2023) | Various (see B.5) |
| Gender Development Index (subnational) | UNDP subnational HDR | Varies by country | 5 April 2024 |

**B.2 Country Sample and Inclusion Criteria**

- **Initial sample:** All 194 WHO member states.
- **Inclusion criteria for panel models:** At least 10 years of non‑missing data for the outcome variable between 2000‑2021.
- **Final balanced panel (HALE models):** 167 countries.
- **Unbalanced panels (other outcomes):** Sample sizes as reported in Tables 3 and 4.
- **Excluded countries:** Small island states with persistent missing data (e.g., Nauru, Tuvalu, Palau) and countries with incomplete vital registration affecting HALE estimation (e.g., Somalia, South Sudan for early years). Complete exclusion list is provided in the replication code (exclusion_do file).

**B.3 Handling of Missing Data**

**B.3.1 Covariates (GDP, education, urbanization, health expenditure, workforce density)**

| Gap Length | Handling Method | Rationale |
| --- | --- | --- |
| ≤3 consecutive years | Linear interpolation | Assumes smooth temporal change; avoids listwise deletion |
| >3 consecutive years | No interpolation; observations excluded | Excessive uncertainty; interpolation would be arbitrary |
| Intermittent missing (single year) | Linear interpolation using adjacent years | Standard practice in panel econometrics |

**B.3.2 Outcome variables (HALE, U5MR, MMR, UHC SCI, DTP3, catastrophic spending)**

- **No imputation performed.** Missing outcome years are excluded from estimation.
- For catastrophic spending (biennial data), values are assigned to both years of the two‑year period (e.g., 2005 value used for 2005 and 2006).

**B.3.3 Service indicators (SBA, ANC4)**

- Interpolated linearly between survey years (typically every 3‑5 years).
- No extrapolation before first survey or after last survey.

**B.3.4 GAVI disbursements**

- Zero values assigned for years before country became GAVI‑eligible.
- No interpolation for gaps in reported disbursements (treated as missing).

**B.4 Definitional Breaks and Harmonization**

| Variable | Break Year | Issue | Harmonization Procedure |
| --- | --- | --- | --- |
| Health expenditure | 2011 | SHA 2011 replaced SHA 2003 | Ratio adjustment: calculated country‑specific ratio of SHA 2011 to SHA 2003 in 2011; applied backward to 2000‑2010 |
| Education (female secondary) | 2011 | ISCED 2011 replaced ISCED 1997 | UNESCO provides concordance tables; mapped to “upper secondary and above” consistently |
| Urban population | Varies by country | National definitions of “urban” changed | Used UN WUP correction factors (provided in repository) to back‑adjust |
| GDP per capita (PPP) | 2017 | ICP 2017 benchmark | World Bank data already expressed in constant 2017 international dollars; no additional adjustment needed |
| Health workforce density | 2010 | WHO revised estimation methods for physicians, nurses, midwives | Used break‑adjusted series provided by WHO (flag variable included) |

**B.5 DHS/MICS Data Processing (for SII Estimation)**

**B.5.1 Survey inclusion criteria**

- Surveys conducted between 2014 and 2023.
- Children aged 12‑23 months at time of survey.
- Complete information on DTP3 vaccination status (card + mother recall).
- Valid wealth index variable (quintiles or continuous score).
- Subnational region identifier.

**B.5.2 Wealth index harmonization**

- DHS: used original wealth index scores (continuous) and quintiles.
- MICS: used continuous wealth score when available; otherwise quintiles converted to fractional rank using midpoint method.
- **Fractional rank calculation** for logistic SII regression:

$$R_{i}=\frac{1}{N}\sum_{j=1}^{i} w_{j}-\frac{w_{i}}{2}$$

where $w_{j}$ is the sampling weight for household $j$, sorted by wealth score.

**B.5.3 Covariate standardization across surveys**

| Covariate | DHS Definition | MICS Definition | Harmonized Definition |
| --- | --- | --- | --- |
| Child age | Months (0‑59) | Months (0‑59) | Used as continuous; restricted to 12‑23 months for DTP3 |
| Child sex | 1=male, 2=female | 1=male, 2=female | Recoded as 0=male, 1=female |
| Maternal education | None, primary, secondary, higher | None, primary, secondary, higher | Collapsed to binary: secondary or higher = 1 |
| Urban/rural | Urban = 1 | Urban = 1 | No change |
| Subnational region | Admin‑1 (state/province) | Admin‑1 | Standardized names using UN mapping tables |

**B.5.4 Survey weights**

- Applied using svy commands in Stata with primary sampling unit (PSU) and strata.
- For bootstrap SII standard errors: cluster resampling at PSU level with 1,000 replications.

**B.6 Variable Transformations**

| Variable | Transformation | Formula | Rationale |
| --- | --- | --- | --- |
| GDP per capita | Natural log | ln(GDPpc) | Right‑skewed; elasticity interpretation |
| U5MR | Natural log | ln(U5MR) | Right‑skewed; approximate normality |
| MMR | Natural log | ln(MMR) | Right‑skewed; variance stabilization |
| HALE | None (years) | — | Already approximately normal |
| UHC SCI | None (0‑100) | — | Bounded; linear model used |
| DTP3 coverage | None (0‑100) | — | Bounded; linear model used |
| Catastrophic spending | None (0‑100) | — | Percentage point interpretation |
| Health expenditure | % of GDP | — | Level interpretation |
| Female education | Percentage points | — | Level interpretation |
| Urbanization | Percentage points | — | Level interpretation |
| Health workforce density | Per 10,000 population | — | Level interpretation |

**B.7 Merging and Panel Construction**

**B.7.1 Merge keys**

- Primary key: ISO3 country code + year (YYYY).
- Country codes harmonized to ISO3 standard across all sources (mapping table provided in repository).

**B.7.2 Merge order**

1. Start with master country‑year frame (all country‑year combinations for 2000‑2021).
2. Merge HALE, U5MR, MMR, DTP3 (outcomes).
3. Merge covariates (GDP, education, urbanization, health expenditure, workforce density).
4. Merge service indicators (SBA, ANC4).
5. Merge UHC SCI and catastrophic spending.
6. Merge GAVI disbursements.
7. Merge mandatory prepayment system indicator.

**B.7.3 Final dataset structure**

- Format: long panel (country‑year).
- Balanced for HALE models (167 countries × 20 years = 3,340 observations; actual = 3,276 after missing outcomes).
- Unbalanced for other outcomes (as reported).

**B.8 Code Organization and Execution**

| File Name | Purpose | Dependencies |
| --- | --- | --- |
| 00_master.do | Runs all scripts in order | None |
| 01_import_raw.do | Imports raw data from CSV/Excel files | Raw data in /raw/ |
| 02_harmonize.do | Applies definitional break adjustments, harmonization | 01_import_raw.do |
| 03_interpolate.do | Linear interpolation for covariates | 02_harmonize.do |
| 04_merge_panel.do | Creates final country‑year panel | 03_interpolate.do |
| 05_decomposition.R | Continuous‑change HALE decomposition | WHO GHE data |
| 06_panel_fe.do | Fixed‑effects regression models | 04_merge_panel.do |
| 07_sii.do | SII estimation from DHS/MICS | DHS/MICS data |
| 08_gdi_correlation.do | Subnational GDI‑DTP3 correlation | Subnational HDR data |
| 09_projections.do | Dynamic scenario projections | 06_panel_fe.do output |
| 10_figures.R | Generates all figures | All analysis outputs |
| appendix_table_a1.do | Robustness checks (Driscoll‑Kraay, GMM) | 04_merge_panel.do |

**Execution instructions:**

bash

*# Stata (run from repository root)*

stata -b do 00_master.do

*# R (run from repository root)*

Rscript 10_figures.R

All intermediate datasets are saved in /output/ with clear versioning. The complete workflow runs in approximately 45 minutes on a standard desktop computer (16 GB RAM)
